# Lipid-Directed Covalent Labeling of Plasma Membranes for Long-Term Imaging, Barcoding and Manipulation of Cells

**DOI:** 10.1021/jacsau.4c01134

**Published:** 2025-02-12

**Authors:** Nathan Aknine, Remi Pelletier, Andrey S. Klymchenko

**Affiliations:** Laboratoire de Bioimagerie et Pathologies, UMR 7021 CNRS, ITI SysChem-Chimie des Systèmes Complexes, Faculté de Pharmacie, Université de Strasbourg, 67401 Illkirch, France

**Keywords:** fluorescent membrane probes, directed covalent labeling, cellular imaging, cell tracking, cell surface engineering

## Abstract

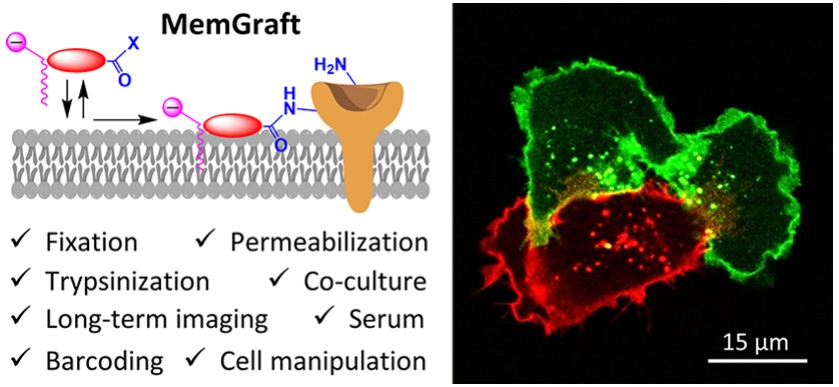

Fluorescent probes for cell plasma membranes (PM) generally exploit a noncovalent labeling mechanism, which constitutes a fundamental limitation in multiple bioimaging applications. Here, we report a concept of lipid-directed covalent labeling of PM, which exploits transient binding to the lipid membrane surface generating a high local dye concentration, thus favoring covalent ligation to random proximal membrane proteins. This concept yielded fluorescent probes for PM called MemGraft, which are built of a dye (cyanine Cy3 or Cy5) bearing a low-affinity membrane anchor and a reactive group: an activated ester or a maleimide. In contrast to specially designed control dyes and commercial Cy3-based labels of amino or thiol groups, MemGraft probes stain efficiently PM, revealing the crucial role of the membrane anchor combined with optimal reactivity of the activated ester or the maleimide. MemGraft probes overcome existing limitations of noncovalent probes, which makes them compatible with cell fixation, permeabilization, trypsinization, and the presence of serum. The latter allows long-term cell tracking and video imaging of cell PM dynamics without the signs of phototoxicity. The covalent strategy also enables staining and long-term tracking of cocultured cells labeled in different colors without exchange of probes. Moreover, the combination of MemGraft-Cy3 and MemGraft-Cy5 probes at different ratios enabled long-term cell barcoding in at least 5 color codes, important for tracking and visualizing multiple populations of cells. Ultimately, we found that the MemGraft strategy enables efficient biotinylation of the cell surface, opening the path to cell surface engineering and cell manipulation.

## Introduction

Cell plasma membrane (PM) is an indispensable frontier at the cell surface. It is not a simple barrier between the cell interior and the environment, but a highly organized two-dimensional structure with specially organized lipids and proteins, which plays essential roles in the signaling, transport of nutrients, cell–cell recognition, motility, etc.^1−3^ Therefore, the development of fluorescent tools to image and probe cell plasma membranes is currently a subject of intensive research.^4−7^

So far, a large variety of fluorescent membrane probes have been developed. Some operate like basic membrane markers, which include for instance cyanine derivatives, like DiO, DiI, PKH, and more advanced analogues, such as MemBright.^8,9^ Other probes are designed for sensing local membrane properties, including local polarity,^10−12^ viscosity,^13,14^ and tension^15−17^ that help to understand lipid organization, or detect local pH,^18^ ions,^19^ specific lipids^20,21^ or membrane proteins.^22,23^ However, most of those examples are based on lipophilic or amphiphilic lipid anchors or protein ligands, which interact noncovalently with cell plasma membranes. The noncovalent labeling of PM implies that the probes could exchange with the environment, in particular, membranes of other cells or serum proteins. Moreover, after fixation and permeabilization, used in a variety of staining protocols especially immunofluorescence,^24,25^ these probes are generally washed away from the cell surface, because membrane integrity is compromised and most of the lipids and lipid probes are removed by the detergents. These issues make those probes also incompatible with expansion microscopy,^26−28^ tissue clearing^29−31^ and correlative light and electron microscopy (CLEM).^32,33^ Moreover, fast lateral diffusion of PM probes makes them poorly compatible with super-resolution imaging, in particular single-molecule localization microscopy.^34−38^

A promising solution is the covalent grafting of fluorescent dyes to proteins, which is compatible with both fixation and permeabilization protocols. The efficient labeling approaches include the use of potent tags, such as SnapTag,^39,40^ HaloTag,^41,42^ ClipTag,^43^ etc., but they require genetic modification of the cell. Direct labeling of native proteins on the cell surface could be done, for instance, by activated esters of *N*-hydroxysuccinimide (NHS). However, this approach is not so convenient and efficient, because this labeling reaction requires high concentrations of the labeling agent (∼5 μM).^44^ Therefore, we hypothesized that the labeling could be accelerated by designing a reactive probe capable of first binding to the cell surface noncovalently. Then, the high local concentration of the probe would favor fast labeling of neighboring proteins. In this respect, one should mention affinity-based protein labeling,^45,46^ where an affinity ligand that binds a specific protein site favors covalent grafting to a proximal reactive group of the protein. It was successfully applied as covalent inhibitors in drug discovery,^47,48^ as well as for site specific labeling of antibodies^45,49,50^ and specific labeling of membrane receptors.^51−54^ However, these are approaches for selective protein labeling, which would target only a limited fraction of specific proteins, inefficient for bright staining of PM. Therefore, we considered a different strategy where the designed probe would label nonselectively a large variety of membrane proteins. For this purpose, noncovalent interaction with lipids of PM could be exploited. The abundance of lipids on the PM would ensure that membrane-bound probes would randomly label any available membrane protein, which could provide efficient high density covalent plasma membrane labeling.

Although the targeting of PM is usually done with high-affinity membrane probes,^4−7^ the removal of nonreacted probes after the covalent labeling would be difficult in this case. Therefore, it would be interesting to explore low-affinity membrane targeting groups, which could reversibly bind to cell plasma membranes. Recently, we introduced low-affinity amphiphilic anchors for plasma membrane targeting composed of butyl chain and charged sulfonate headgroup.^11,38^ Reversible low-affinity binding of probes based on solvatochromic Nile Red^11^ and fluorogenic dimer^38^ enabled PAINT-type super-resolution imaging of plasma membranes. However, it remains unexplored whether this reversible binding could favor a reaction between the activated ester or maleimide group and membrane proteins, which would enable efficient covalent grafting of fluorescent probes to plasma membranes.

In the present work, we designed a series of cyanine derivatives bearing a low-affinity membrane targeting anchor group in combination with a reactive group for conjugation. We found that the combination of low-affinity anchor with the activated ester or maleimide group provides strong labeling of the cell surface for both cyanine 3 (Cy3) and cyanine 5 (Cy5) analogues, which was not the case for nonactivated carboxylate control analogues. Moreover, the control compound with a truncated anchor (without butyl group) did not show plasma membrane labeling, which highlights the fact that membrane protein labeling is catalyzed by probe-lipid interactions. Importantly, the obtained plasma membrane labeling showed resistance to the presence of serum as well as to cell fixation and permeabilization in contrast to a reference plasma membrane probe of MemBright family. The MemGraft strategy also enables long-term cell tracking and multicolor barcoding of multiple cocultured cell populations. The covalent strategy also allowed design of the MemGraft probe for cell biotinylation and further cell manipulation with magnetic beads. The developed probe will find multiple applications in cellular imaging, cell surface engineering, and cell manipulation, where robust grafting to the cell surface is needed.

## Materials and Methods

### General Methods and Materials

All of the reagents were purchased from Sigma-Aldrich, Alfa Aesar, or TCI and used without any purification. Cy3 and Cy5 diacids, 3-(butylammonio)propane-1-sulfonate were synthesized as described previously.^11,38^ Disulfo-Cy3 NHS ester was purchased from Tebu-Bio. Disulfo-Cy3-maleimide (disulfo-Cy3-Mal) was purchased from Interchim. MemBright-488 (MemGlow-488 by Cytoskeleton) was synthesized as described before.^9^ Milli-Q-water (Millipore) was used in all experiments. NMR spectra were recorded on a BrukerAvance III 400 MHz spectrometer and a BrukerAvance III 500 MHz spectrometer. Mass spectra were obtained using an Agilent Q-TOF 6520 mass spectrometer with electrospray ionization and a Bruker Microflex LRF system for MALDI-TOF MS. Absorption and emission spectra were recorded on an Agilent Cary 5000 UV–vis–NIR spectrophotometer and an Edinburgh FS5 spectrofluorometer, correspondingly. Synthesis of MemGraft-Cy3, MemGraft-Cy5, MemGraft-Cy3M and other dyes is described in Supporting Information.

### Cell Lines, Culture Conditions, and Treatment

U87 (ATCC HTB-14) cells and Hela (ATCC CCL-2) cells were grown in Eagle’s Minimum essential medium (EMEM, Gibco-Invitrogen), supplemented with 10% fetal bovine serum (FBS, Lonza), 2 mM l-glutamine (Gibco-Invitrogen), 1% nonessential amino acid solution (Gibco-Invitrogen) and sodium pyruvate 1 mmol/L at 37 °C in a humidified 5% CO_2_ atmosphere. Cells were seeded onto a 35-mm chambered coverglass (IBIDI) at a density of 5 × 10^4^ cells/well 24 h before microscopy measurement.

For microscopy imaging, the attached live cells in IBIDI dishes were washed once with warm Hanks’ balanced salt solution (HBSS, Gibco-Invitrogen). Then, an aliquot of a stock solution of a corresponding dye (a MemGraft probe or a control) in DMSO was diluted into 1 mL of HBSS (to 0.1–1 μM final concentration), and the obtained mixture was rapidly added to the cells and incubated for 5 min at room temperature. Then, the attached cells in IBIDI dishes were washed twice with HBSS before imaging. Serum resistance experiments were performed with the same protocol, followed by the incubation of the cells with 20% FBS in phosphate-buffered saline (PBS). For fixation and permeabilization experiments, the dye solution was removed from the cells, and the attached cells were washed twice with HBSS and incubated for 15 min with 4% (w/v) formaldehyde solution in PBS at room temperature. The fixative solution was removed, and the cells were washed by pipetting PBS against the side of the dish 3 times. Then, the cells were incubated with 1 mL of 0.1% Tween-20 in PBS for 12 min at room temperature. The permeabilization solution was removed and washed 3 times with PBS before imaging.

For coculture experiments, the cells were seeded in a 6-well plate at a density of 5 × 10^4^ cells/well 24 h before the experiment. The attached cells were stained by MemGraft-Cy3 or MemGraft-Cy5 as described above, washed twice with HBSS, detached with StemPro Accutase by incubating for 10 min at 37 °C, centrifuged, and distributed in IBIDI dishes filled with the culture medium described previously. After 5 or 29 h of incubation, the cocultured cells were imaged in the culture medium without more treatment. Cell barcoding was done using the same protocol but using mixtures of MemGraft-Cy3 and MemGraft-Cy5 at different molar ratios (total concentration was 1 μM). The mixed barcoded cell populations were coincubated for 3 h at 37 °C before imaging.

In the experiments on blocking amino-group sites of membrane proteins, the U87 cells were treated with Sulfo-NHS-Acetate (Pierce^TM^ ThermoFisher) at 1 mM in HBSS for 60 min at 37 °C. The blocking solution was removed, and the cells were washed with PBS and stained with the corresponding dye solution following the previously described protocol.

### Fluorescence Microscopy

Confocal imaging of cells was performed on a Leica TCS SP8 confocal microscope with a HCX PL APO 63×/1.40 OIL CS2 objective and two 12-bit photomultipliers. The excitation light was provided by lasers at 488, 552, and 638 nm for MemBright-488, MemGraft-Cy3, and MemGraft-Cy5, respectively. The fluorescence from these three probes was detected at the corresponding spectral ranges: 500–540 nm, 570–620 nm, and 650–700 nm. Imaging of barcoded cells was done using the same microscopy settings as for cells labeled individually by MemGraft-Cy3 and MemGraft-Cy5. All of the parameters at each channel were left constant; the illumination power was adjusted to achieve a good signal for each probe. When the performances of the probes were compared, all instrumental conditions were fixed. All of the images were processed using ImageJ software.

Video microscopy experiments on live cells were performed on a Leica DMIRE 2 microscope with a HXC PL APO 40*×*/1.25 oil objective equipped with a Photometric Prime camera. The sample chamber is controlled by a thermostatic chamber and a CO_2_-controlled incubation chamber for long-term live imaging conditions. The system is piloted by the software Metamorph version 7.8.13.0 from Molecular Devices. The excitation light is provided by the LED light source Lumencor SOLA III equipped for exciting light in 5 different channels (DAPI, GFP, Cy3, Cy5 and transmitted light). The images were processed and converted into videos using ImageJ software.

### Flow Cytometry Analysis

U87 cells were seeded at a density in 1 × 10^6^/well in a 6-well plate 24 h before flow cytometry measurement. For cytometry analysis, the attached live cells were washed once with warm Hanks’ balanced salt solution (HBSS, Gibco-Invitrogen). After that, 5 mL of a corresponding dye (MemGraft-Cy3 or disulfo-Cy3-NHS) solution at 0.1 and 1 μM in HBSS was added, and the cells were incubated for 10 min at room temperature. Then, the attached cells in IBIDI dishes were washed twice with HBSS. The cells were then detached with StemPro Accutase incubation for 10 min at 37 °C, centrifuged, and resuspended in a solution of 1% BSA in PBS. This suspension was passed through a 50 μm nylon mesh filter. For the experiment, the cells were analyzed using a MACSQuant VYB flow cytometer. MemGraft-Cy3 and commercial disulfoCy3-NHS were excited with a 561 nm laser, and the fluorescence was collected at 579–593 nm (PE and SYTOX orange filter, named Y1). The detectors were calibrated by light scattering and fluorescence using MACSQuant fluorescent calibration beads.

### Magnetic Cell Manipulation

U87 cells were seeded at a density in 1 × 10^6^/well 24h before the experiment. The attached live cells were washed once with PBS. After that, 2 mL of MemGraft-Cy3-Biotin solution at 1 μM concentration in HBSS was added in 3 wells and MemGraft-Cy5 solution at 1 μM in HBSS was added in 3 other wells and were incubated for 10 min at room temperature. Then, all the attached stained cells in dishes were washed with PBS and detached by 5 min of incubation at 37 °C with an Accutase solution, and the cells with same staining were mixed together. The suspensions of cells were centrifuged for 5 min at 1.500 rpm. The supernatant was discarded, and the cells were resuspended in OptiMEM. The two batches of cells stained with MemGraft-Cy5 and MemGraft-Cy3-Biotin were mixed together by pipetting up-and-down. One IBIDI dish was reseeded in OptiMEM with this suspension mixture as a negative control. The rest of the suspension was mixed with 3 times prewashed Pierce Streptavidin Magnetic Beads from Thermo Fisher (because of toxic azide in the formulation), distributed in 0.5 mL centrifuge tubes, and placed on a magnetic separation stand from Promega. After 2 min, the supernatant containing a part of the cells was carefully collected and the cells were seeded in a new IBIDI dish in OptiMEM. The rest of the cells were taken out from the magnetic separation stand, suspended in OptiMEM, and reseeded in a new IBIDI dish. The 3 dishes were incubated for adhesion for 3 h at 37 °C before the imaging.

## Results

### Design and Synthesis

**Figure 1:**
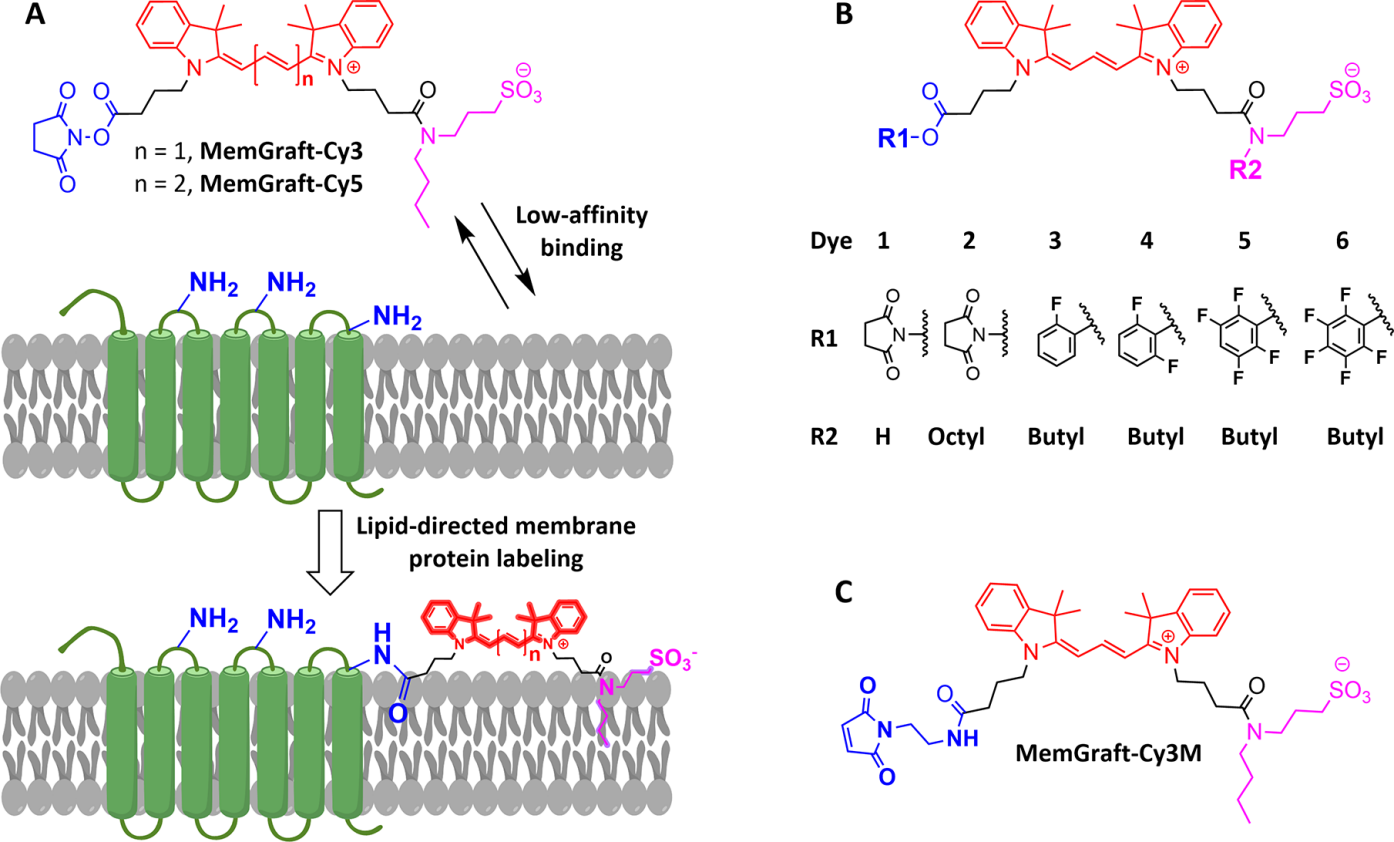
MemGraft probes and the concept of lipid-directed covalent labeling of plasma membranes. Chemical structures of MemGraft-Cy3 and MemGraft-Cy5 probes (A), control dyes with activated ester (B) and MemGraft-Cy3M probe (C).

The design of the probes is based on a cyanine fluorophore, bearing on one end a low-affinity membrane anchor composed of a butyl chain and sulfonate group, while the other end of cyanine bears an activated ester (Figure 1A, B). In our hypothesis, the low-affinity membrane anchor would provide a transient binding to a lipid membrane, as we showed for Nile Red^11^ and cyanine dimers,^38^ which is expected to ensure a high local concentration of the probe at the membrane level (vs buffer) in close proximity to membrane proteins (Figure 1). The latter should favor covalent grafting of the active ester with amino groups of membrane proteins, which is needed for covalent labeling of plasma membranes (Figure 1). The synthesis was based on our previous report,^38^ where corresponding cyanine (Cy3 or Cy5) dicarboxylate, was first monofunctionalized with the anchor (3-(butylammonio)propane-1-sulfonate) and then, after purification, the second carboxylate was converted into activated NHS ester using TSTU (Figure S1). These compounds are called here MemGraft-Cy3 and MemGraft-Cy5 probes, respectively (Figure 1). To understand the role of the anchor group, we synthesized Cy3 analogues without butyl chain (dye **1**) and with octyl chain (dye **2**). To this end, the Cy3 dicarboxylate was first monofunctionalized by 3-aminopropane-1-sulfonate or 3-(octylammonio)propane-1-sulfonate, and the second carboxylate was converted into NHS ester using TSTU to yield dyes **1** and **2**, respectively. Then, to explore the importance of the activated ester, we also synthesized MemGraft-Cy3 analogues, bearing fluorophenyl activated esters of increasing reactivity (Figure 1): 2-fluorophenyl (dye **3**) and 2,6-difluorophenyl (dye **4**), 2,3,5,6-tetrafluorophenyl (dye **5**) and 2,3,4,5,6-pentafluorophenyl (dye **6**), which are also expected to react with amino groups of proteins.^55^ The nonactivated control compounds for MemGraft-Cy3 and MemGraft-Cy5 probes were intermediate carboxylic acid analogues: dyes **7** and **8**, respectively. Moreover, to expand the approach to another type of bioconjugation, we replaced an activated ester with a maleimide group (MemGraft-Cy3M), which is expected to react with the thiol groups of membrane proteins.

### Labeling of Cell Plasma Membranes

**Figure 2:**
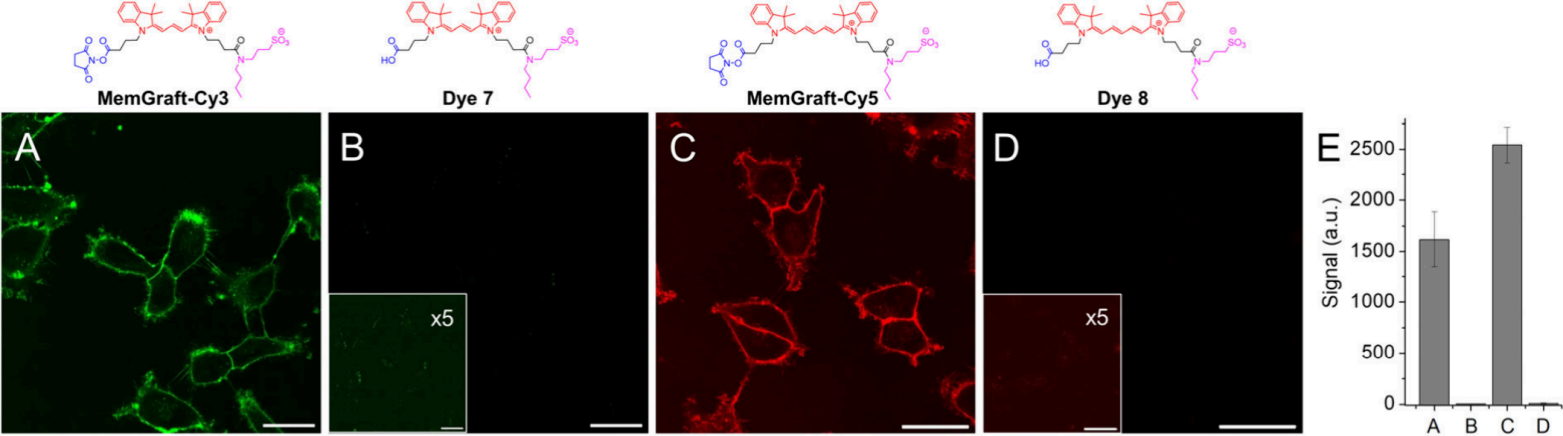
Fluorescence labeling of plasma membranes using MemGraft-Cy3 (A) and MemGraft-Cy5 (C) dyes and their nonactivated carboxylic acid analogues **7** (B) and **8** (D). Confocal fluorescence microscopy of U87 cells incubated for 5 min with the dyes at a 1 μM concentration. Scale bar: 30 μm. The insets of parts B and D are the same images with 5-fold multiplied intensity to show the lack of labeling. The larger images with contours highlighting position of the cells are shown in Figure S4. (E) Quantitative image analysis: fluorescence signal at the plasma membrane (fluorescence intensity minus background intensity) for the conditions of panels A-D, respectively. Four regions of interest were analyzed per condition. The errors are the standard deviations of the mean.

**Figure 3:**
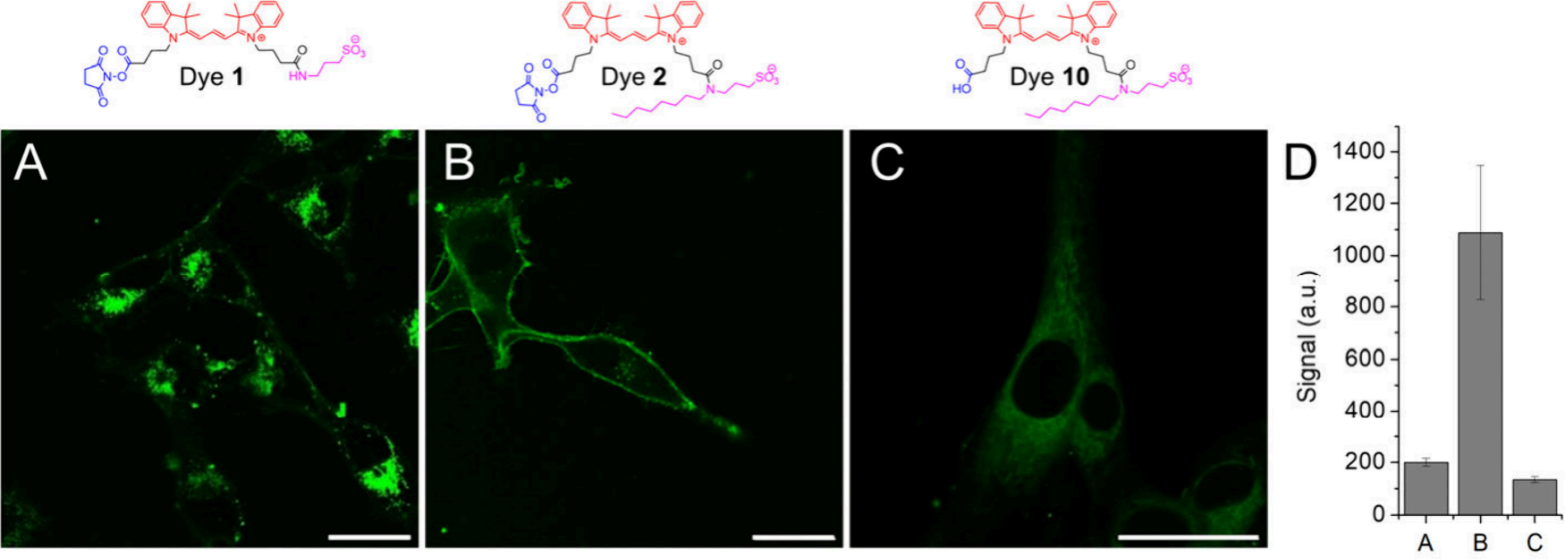
Role of the membrane anchor in analogues of MemGraft-Cy3. Confocal fluorescence microscopy of U87 cells incubated with dyes **1** (A), **2** (B) and **10** (C). Dye concentration was 1 μM. Scale bar: 30 μm. (D) Quantitative image analysis: fluorescence signal at the plasma membrane (fluorescence intensity minus background intensity) for the conditions of panels A-C, respectively. Four regions of interest were analyzed per condition. The errors are the standard deviation of the mean.

First, we characterized the most reactive activated ester (NHS) derivatives. The Cy3 and Cy5 probes (MemGraft-Cy3, and MemGraft-Cy5, respectively) were incubated with live cells for 5 min and then imaged after washing. A clear cell surface fluorescence was observed for both Cy3- and Cy5-based probes in U87 cells (Figure 2). Imaging experiments on another cell line (HeLa) with MemGraft-Cy3 showed similar cell surface labeling (Figure S2). The staining profiles of MemGraft-Cy3 and MemGraft-Cy5 were nearly identical to that of a reference probe MemBright-488^9^ (Figure S3), suggesting efficient PM targeting. At the same labeling conditions, control analogues without activated carboxylate (dyes **7** and **8**) showed practically no membrane staining (Figures 2 and S4). The latter suggests that, as expected, these control probes exhibit low affinity to plasma membranes and therefore can be completely removed by washing. We also compared MemGraft-Cy3 with its methyl ester analogue (dye **17**), which cannot react with the amino groups. Importantly, no plasma membrane labeling was observed (Figure S5). Given that the structure and calculated LogP (cLogP) of these two control dyes (cLogP is 0.82 and 1.25 for dyes **7** and **17**, respectively) are very close to those of MemGraft-Cy3 (cLogP = 0.52), we can conclude that, similar to these two controls, MemGraft-Cy3 does not present any significant fraction of noncovalently bound species to the cell surface after labeling and washing steps. Therefore, the signal from the activated probes at the plasma membrane corresponds exclusively to that of the covalently grafted probes. To understand the role of hydrophobic interactions of the probes in membrane protein labeling, we varied the alkyl chain of the anchor of MemGraft-Cy3. Thus, we designed an analogue without any alkyl group (dye **1**) and with an octyl group (dye **2**), which are expected to strongly alter their affinity to lipid membranes compared to the parent MemGraft-Cy3. Remarkably, control probe **1** showed mainly intracellular fluorescence with poor plasma membrane labeling, in contrast to MemGraft-Cy3 (Figure 3). This dramatic change in the labeling profile caused by the removal of the butyl group indicates that the latter plays a crucial role in PM labeling. On the other hand, dye **2** with octyl chain showed good PM labeling, in contrast to its nonactivated acid analogue **10** (Figure 3). Indeed, the butyl or octyl groups should favor transient binding and certain accumulation of the probe on the lipid membrane surface.^11^ This is expected to ensure a high local concentration of the MemGraft probes that would favor covalent grafting to membrane proteins (Figure 1). On the other hand, the analogue without the butyl group cannot stay sufficiently long at the cell surface and show some capacity to penetrate PM, which can explain the lack of PM labeling and the significant intracellular signal. A similar difference between MemGraft-Cy3 and the control dye **1** without a butyl group was observed at a lower concentration (0.1 μM, Figure S6), which additionally shows that the MemGraft probe can efficiently label PM despite this low dye concentration.

Then, we tested control dyes **3**-**6**, which bear activated esters for amino group labeling of varied reactivity. However, they did not provide efficient plasma membrane labeling but rather significant intracellular staining (Figure S7). Nevertheless, we observed an interesting tendency. While compounds **3** and **4** with lower reactivity toward amino groups showed exclusively intracellular staining, more reactive dyes **5** and **6** exhibited a clearly seen cell contour (Figure S7). The membrane staining was the best seen for the most reactive dye **6** in this series, where membrane extensions could be well identified, although the intracellular fluorescence remained strong (Figure S7). These results indicate that reactivity of the active ester is crucial for efficient plasma membrane labeling. We can conclude that, in contrast to NHS esters, none of the fluorophenyl esters are reactive enough, as the internalization goes faster than the protein labeling. Moreover, fluorophenyl esters are much more hydrophobic than the NHS is, which could additionally favor internalization by crossing the plasma membrane.

**Figure 4:**
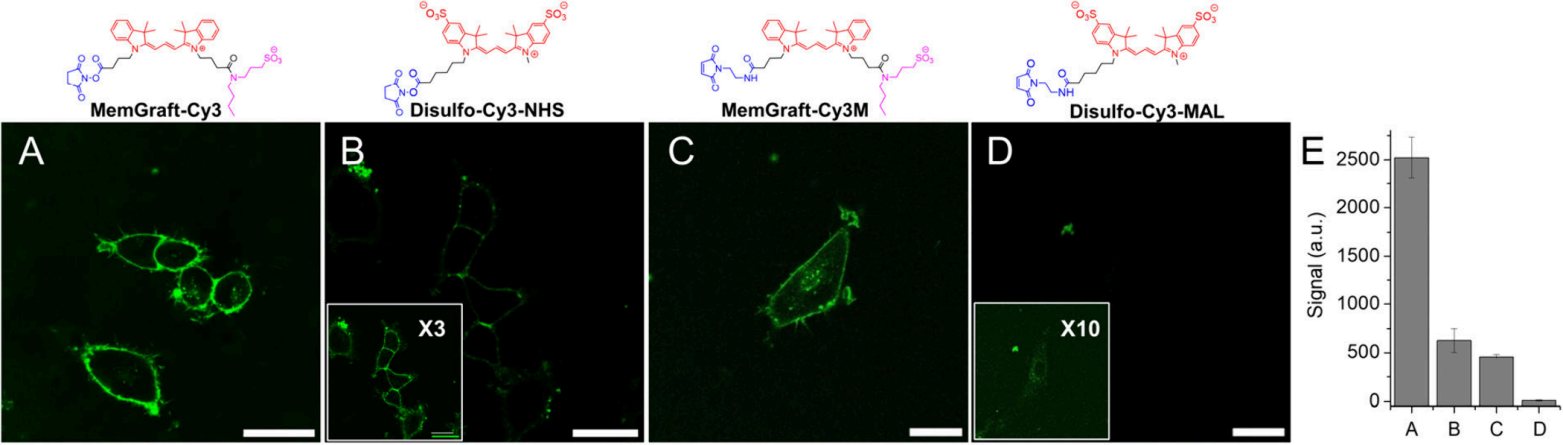
Comparison of MemGraft-Cy3 and MemGraft-Cy3M with commercial labels of close structure. (A, B) Confocal images of U87 cells incubated with MemGraft-Cy3 (A) and disulfo-Cy3-NHS (B) at 100 nM concentration. Scale bar: 30 μm. (C, D) Confocal images of U87 cells incubated with MemGraft-Cy3M (C) and disulfo-Cy3-MAL (D) at a 500 nM concentration. Scale bar: 30 μm. (E) Quantitative image analysis: fluorescence signal at the plasma membrane (fluorescence intensity minus background intensity) for the conditions of panels A-D, respectively. Four regions of interest were analyzed per condition. The errors are the standard deviation of the mean.

We also compared our MemGraft probes with commercial reference dye disulfo-Cy3-NHS, which is routinely used for amino-group labeling of proteins. Fluorescence microscopy of cells labeled with disulfo-Cy3-NHS at the same experimental conditions showed a significantly smaller signal at the cell surface compared to MemGraft-Cy3 (Figure 4A,B). Quantitative image analysis revealed ∼4-fold higher signal from cell PM labeled with MemGraft-Cy3 compared to disulfo-Cy3-NHS (Figure 4E). This result confirms that the presence of membrane anchor significantly improves membrane labeling, because it favors higher local dye concentrations in the membrane that can catalyze the reaction with the amino groups of membrane proteins. To further shed light on the nature of the labeling, the amino residues on the cell surface were blocked by treatment with Sulfo-NHS-acetate on cells.^56^ This is a highly polar agent, which is expected to acetylate only water-exposed amino-group residues at the cell surface. After the treatment, the cells were stained with MemGraft-Cy3 or disulfo-Cy3-NHS and the fluorescence intensity of the cells was compared to those without the blocking treatment (Figure S8). Remarkably, the blocking by Sulfo-NHS-acetate nearly totally inhibited cell membrane labeling of disulfo-Cy3-NHS with only 8% residual fluorescence signal, while the staining by MemGraft-Cy3 was decreased to a small extent (60% of the residual fluorescence signal). This proves that the nature of the MemGraft labeling is different from classical polar NHS-ester reagents, like disulfo-Cy3-NHS. Due to the presence of the low-affinity membrane anchor, MemGraft-Cy3 probably labels amino groups of proteins in close contact with the lipid bilayer that cannot be accessed by Sulfo-NHS-acetate (Figure S8), while commercial disulfo-Cy3-NHS labels exposed amino groups assessable for Sulfo-NHS-acetate. Then, we also tested maleimide analogue MemGraft-Cy3M and compared it to a commercial thiol label based on Cy3, disulfo-Cy3-MAL. Remarkably, MemGraft-Cy3M showed clear labeling of PM, while disulfo-Cy3-MAL failed to label PM (Figure 4C,D), with an ∼50-fold difference in the signal at the PM level (Figure 4E). This result confirms the crucial role of low-affinity membrane anchor for covalent PM labeling. Disulfo-Cy3-MAL did not label PM at all probably because thiol groups of membrane proteins are not accessible for this highly polar label, while MemGraft-Cy3M, owing to its membrane binding, can reach and react with membrane-shielded thiol groups.

**Figure 5:**
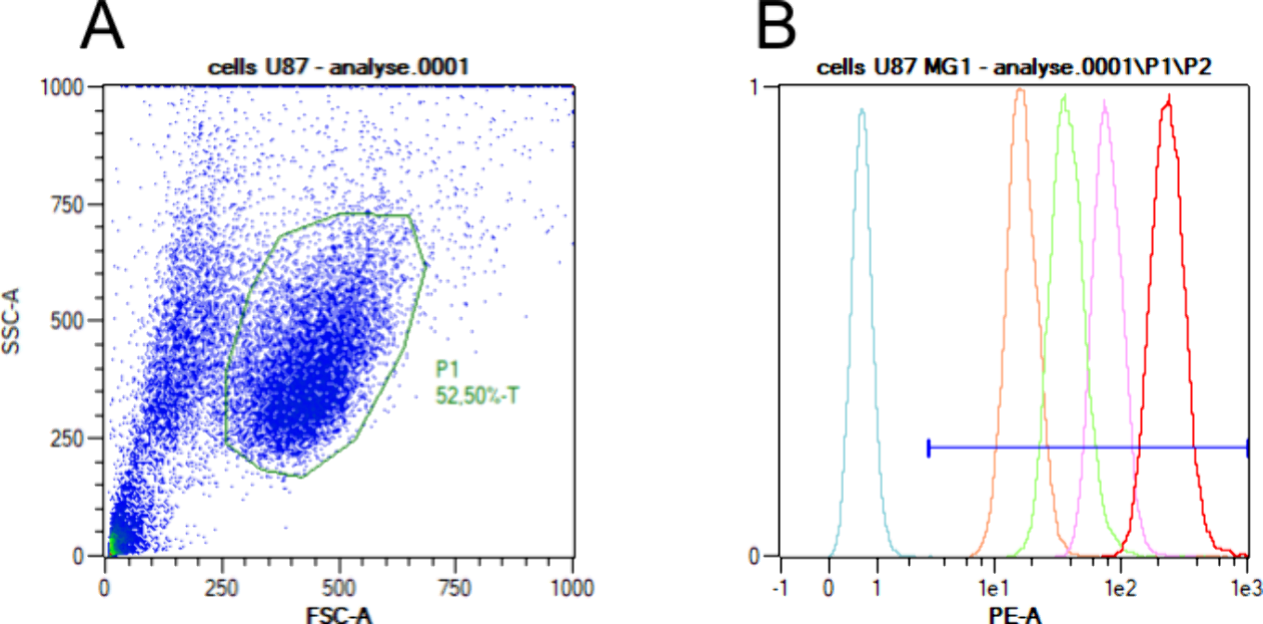
Flow cytometry analysis of MemGraft-Cy3 vs commercial label disulfo-Cy3-NHS. (a) Representative flow cytometry density plot of U87 cells showing side scattered light (granularity) vs forward scattered light (size of cells). The region P1 is established from nontreated cells. Cells doublets are abstracted by filtrating counts out of linearity in FSC-A vs FSC-H plot. (b) Normalized flow cytometry histograms showing cell counts vs fluorescence intensity of not stained cells (blue), cells stained with disulfo-Cy3-NHS 0.1 μM (orange) and 1 μM (pink), or MemGraft-Cy3 at 0.1 μM (green) and 1 μM (red).

The labeling density of MemGraft-Cy3 was evaluated by flow cytometry in comparison to the commercial label disulfo-Cy3-NHS. A population of 25000–60000 single cells was first identified by SSC and FSC analysis, and the fluorescence signals from single cells were then evaluated for both covalent probes at the same concentrations (0.1 or 1 μM). For both studied concentrations, the fluorescence mean intensity of MemGraft-Cy3 probe was 3-foldhigher than that of disulfo-Cy3-NHS (Figure 5), which is in line with the microscopy data (Figure 4). This result suggests a higher density of labeling for MemGraft-Cy3 compared to its analogue without membrane anchor, supported by quantitative microscopy analysis (Figure 4E). Thus, the cytometry data confirmed the superiority of MemGraft approach compared to direct labeling of amino groups by a commercial dye NHS-ester. It highlights the importance of low-affinity membrane anchor in MemGraft that allows the preorganization of the reactive probe close to amino groups of the membrane proteins, required for efficient membrane labeling. It enables relatively high labeling density for rather low probe concentrations from 0.1 to 1 μM.

The conjugation between the MemGraft probe and a model membrane containing surface amino groups was realized to confirm the chemistry of the labeling. Large Unilamellar vesicles (LUVs) were prepared with a lipid composition of DOPC/DOPE (1:1). The formed liposomes present exofacial reactive amino groups on their outer surface, which are expected to react with MemGraft. The liposomes were incubated with a MemGraft-Cy3 probe, followed by purification by size-exclusion column chromatography and further mass spectrometry. We found a mass-to-charge ratio at 1403.58, corresponding to the conjugate of MemGraft-Cy3 with DOPE (Figure S9). This result confirms the capacity of MemGraft-Cy3 to form covalent bonds with amino groups at the membrane surface, which suggests the covalent nature of the labeling in cell PM. However, on the cell surface, the reaction of MemGraft probe with the amino-groups of membrane proteins is more probable, because PE and phosphatidylserine (PS) lipids are lacking at the outer leaflet of PM of healthy cells.^57^ To verify this point, we performed SDS-page on cytosolic and membrane protein extracts of MemGraft-stained cells. The obtained results suggest that a large range of proteins was labeled by MemGraft-Cy3, as it could be seen by a contentious signal over multiple molecular masses from 20 to 250 kDa (Figure S10). In the case of MemGraft-Cy3M, the signal was much weaker but still visible for proteins of different molecular weight, with predominance of one fraction around 50 kDa. In contrast, cytosolic proteins did not show any signal for both MemGraft probes, confirming that the labeling is specific to membrane proteins only. Thus, as expected, chemically reactive MemGraft probes label nonselectively membrane proteins, which is essential for the efficient labeling of cell plasma membranes. Nevertheless, we cannot exclude that some fraction of the MemGraft probe reacts with PE and/or PS lipids of cell PM, especially when their content at the outer leaflet is increased.

### Resistance to Serum

**Figure 6:**
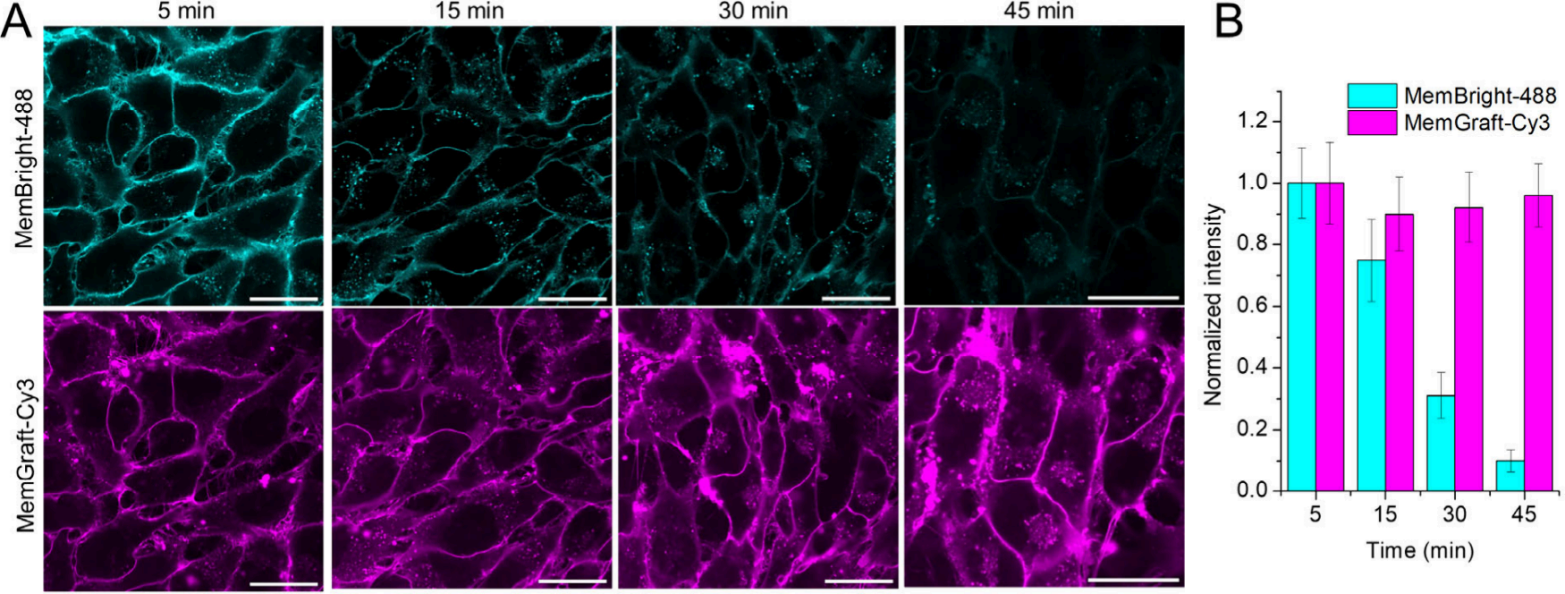
Resistance of a MemGraft probe to the presence of fetal bovine serum (FBS) in the growth medium. (A) Confocal fluorescence microscopy of U87 cells stained with MemGraft-Cy3 (magenta) and reference MemBright-488 probe (cyan) and incubated with 20% FBS for different time. Dye concentrations were 1 μM and 200 nM for MemGraft-Cy3 and MemBright-488, respectively. Scale bar: 30 μm. (B) Quantitative image analysis: fluorescence signal at the plasma membrane (fluorescence intensity minus background intensity) for the conditions of panel A. Four regions of interest were analyzed per condition. The errors are the standard deviation of the mean.

Next, we evaluated the capacity of the new probes to remain on the membrane surface despite the presence of a full growth medium containing serum, which is essential for cell growth. The MemGraft-Cy3 probe was compared to a commercial reference probe (MemBright-488) that binds noncovalently to the plasma membrane.^9^ After 5 min of incubation in 20% FBS, the membrane staining by the MemGraft-Cy3 probe remains similar to that of reference probe MemBright-488 (Figure 6). However, after longer incubation in the presence of serum, the PM signal of MemBright-488 progressively declined and became poorly detectable after 45 min. The remaining signal appeared mainly as perinuclear dotted fluorescence, corresponding probably to endosomes. In contrast, in the presence of 20% FBS, the membrane staining by MemGraft-Cy3 remained similar to that without FBS for the whole observation period, even though a small fraction of intracellular dots could also be observed after 45 min, clearly due to the endocytosis (Figure 6). These differences between the two probes were also confirmed by the quantitative image analysis, suggesting a stable labeling by MemGraft-Cy3 and progressive loss of fluorescence signal by MemBright-488 in the presence of serum (Figure 6B). Thus, we can conclude that FBS can detach MemBright-488 probe from the cell surface, whereas the remaining fraction of the probe is internalized by endocytosis. On the other hand, MemGraft-Cy3 probe resisted well to FBS, so only endocytosis process affected to some extent the imaging contrast over time. Moreover, direct composition of the same sample of cells labeled with MemGraft-Cy3 before and after addition of 20% FBS suggested no significant variations in the signal (Figure S11), which confirms that the labeling is exclusively covalent and the fraction of MemGraft-Cy3 noncovalently bound to PM is negligible. Thus, the MemGraft probe enables imaging of PM in live cells in the presence of serum, which was not possible before with conventional membrane markers. This is important for longitudinal studies of cell behavior without cell starvation.

Therefore, the U87 cells were stained with MemGraft-Cy3 and further studied using long-term imaging in serum-containing medium at 37 °C and 5% CO_2_. Remarkably, we were able to perform continuous video imaging of cells for at least 3 h without significant loss of intensity or cell death (Video S1). These results confirmed high photostability and low phototoxicity of the MemGraft-Cy3 probe, making it a robust tool for long-term imaging of live cells in native conditions of cell culture medium with serum.

### Resistance to Fixation and Permeabilization

**Figure 7:**
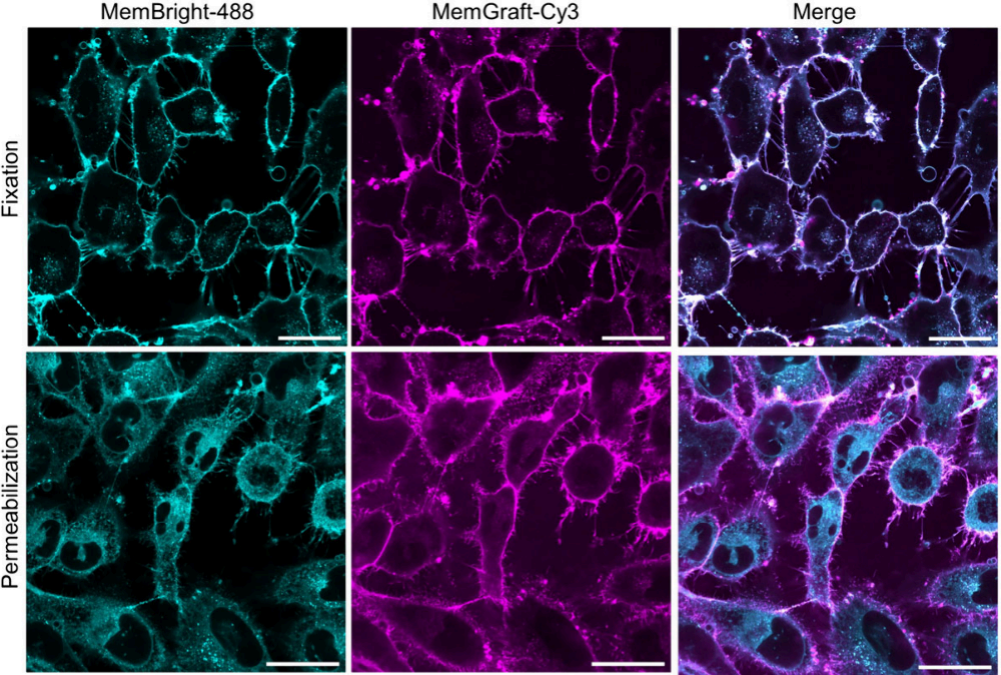
Resistance of a MemGraft probe to fixation with permeabilization. Confocal fluorescence microscopy of U87 cells stained with MemGraft-Cy3 (magenta) and reference MemBright-488 probe (cyan) followed by fixation with PFA (upper panels) and permeabilization with Tween-20 (lower panels). Dye concentrations: 1 μM for MemGraft-Cy3 and 200 nM for MemBright-488. Scale bar: 30 μm.

Even more dramatic effects can be produced on membrane probes during the fixation and permeabilization of the cells. Indeed, while lipids and conventional membrane probes cannot be fixed by paraformaldehyde (PFA),^58,59^ they are expected to be washed out during the permeabilization with surfactant.^60^ Therefore, we studied the effect of fixation and permeabilization on the cell staining with the MemGraft-Cy3 probe in comparison to the reference MemBright-488. After fixation with PFA, both MemBright and MemGraft probes showed plasma membrane staining with good colocalization (Figure 7). This was expected given that the fixation does not directly disturb lipid bilayers and thus the labeling should be preserved even with noncovalent membrane probes. Second, we performed cell permeabilization, which is commonly used for immunofluorescence. Under permeabilization conditions using a standard protocol based on Tween-20 surfactant, MemBright-488 probe lost specificity to plasma membranes, showing the emission signal all over the cells (Figure 7). In contrast, the labeling profile of MemGraft-Cy3 was affected to a minimal extent, showing clear selective plasma membrane staining (Figure 7). Thus, permeabilization based on the detergent treatment led to the removal of lipid bilayers, which led to the loss of plasma membrane staining by MemBright-488, accompanied by internalization of the probe inside the cells. On the other hand, MemGraft-Cy3 probe was attached covalently to membrane proteins, which were fixed by PFA. Then, the application of detergent affected mainly lipids without a strong effect on the fixed membrane proteins, which explains the resistance of the probe to permeabilization.

### Compatibility with Trypsinization and Seeding for Long-Term Observation

Trypsinization is a common method to detach the cells from the surface used in cell seeding and other experiments needed in cell transfer from one surface to another with further long-term observation. As MemGraft probes provide covalent labeling to membrane proteins, we studied whether trypsinization could compromise this labeling. To this end, we incubated the cells with the MemGraft-Cy3 and MemBright-488 probes, then after washing detached and seeded them into another microscopy plate. Remarkably, the MemGraft-Cy3 preserved a strong signal at the plasma membrane after trypsinization, while MemBright-488 did not (Figure S12). The resistance of MemGraft-Cy3 to trypsinization is surprising because one would expect that cleavage of proteins at the cell surface could lead to the removal of the covalent probe. We could explain this notable resistance to trypsinization by its labeling to amino residues of proteins close to lipid membranes, which are probably not assessable to the enzyme, in line with the lack of accessibility observed for the highly polar acetylation reagent sulfo-NHS-acetate as mentioned above.

### Long-Term Cell Tracking

**Figure 8:**
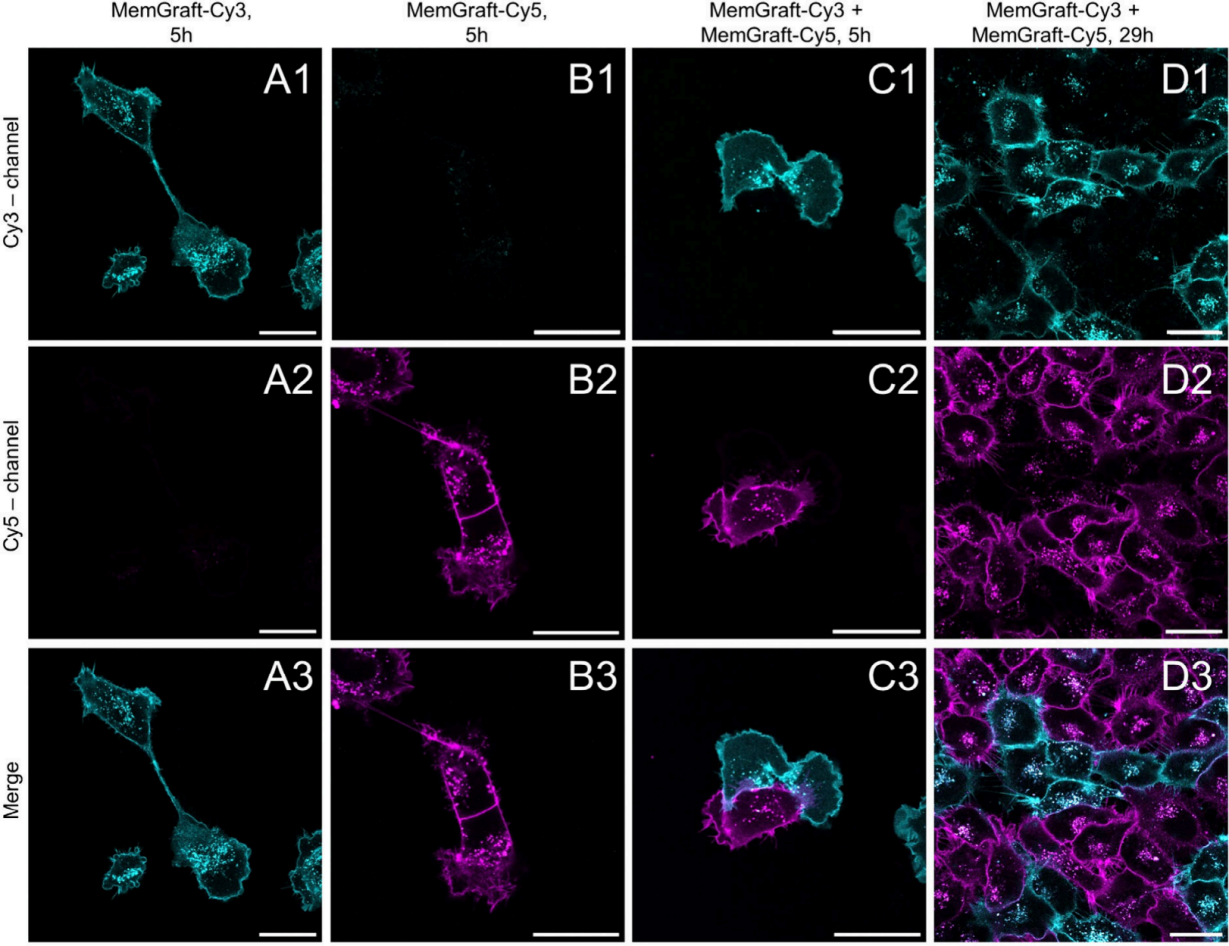
Coculture and confocal fluorescence imaging of cells stained with MemGraft-Cy3 (cyan) and MemGraft-Cy5 (magenta) after 5 h (C) and 29 h (D). Cells stained only with MemGraft-Cy3 (500 nM) or MemGraft-Cy5 (500 nM) are shown, respectively, in A and B panels. Scale bar: 30 μm.

One of the key requirements for the long-term cell experiments is low cytotoxicity of the label. Therefore, we performed a cell viability study for MemGraft-Cy5 probe after 24 h incubation (MemGraft-Cy3 was not studied to avoid a cross-talk with the MTT assay). The results showed negligibly low cytotoxicity of the probe, as >80% of cell viability was observed for concentration range up to 5000 nM without statistically significant effects (Figure S13). Next, we tested the possibility of using MemGraft for tracking cells and their interactions with other cells in a natural growth medium. Classical noncovalent membrane labeling by membrane probes is not suitable for this purpose because serum extracts the probe and the liberated dye could be exchanged between the cells. Two populations of U87 cells were labeled separately with MemGraft-Cy3 and MemGraft-Cy5 and washed. The cells were detached by accutase treatment (milder than trypsinization) and after centrifugation and washing, they were seeded either separately or together in the full-growth medium with FBS. After 5 h of the labeling, both probes showed significant labeling of the cell plasma membrane as well as perinuclear dots, corresponding to endosomes (Figures 8, S14). Thus, as expected, some fraction of the probes internalized, while the remaining fraction stayed at the plasma membrane despite the presence of FBS in the medium. Importantly, MemGraft-Cy3 and MemGraft-Cy5 labeled cells incubated separately were observed exclusively in Cy3 and Cy5 channels, respectively, with minimal cross-talk between the channels (Figure 8-A2-B1), which should allow us to distinguish well the signal from the two different probes. The coincubated cells labeled with two different probes resulted in the observation of two cell populations of different colors (Figure 8-C3-D3). The cells of different colors showed clearly distinctive signals from the individual probes without signs of mixing (Figures 8, S14). It is important to note that the individual colors without mixing were observed even for cells in direct contact with each other (Figure 8-C3). The only exception was the direct contact between the two membranes, which appeared in a different color (Figure 8-C3), because the distance between the two plasma membranes was below the resolution of optical microscopy. This shows that even in the proximity, the probes grafted to the membrane proteins do not exchange with the neighboring cells allowing tracking the interaction of the individual cells. After a 29 h coincubation of two differently labeled cell populations, we could still observe very similar staining profiles, where plasma membranes labeled with MemGraft probes could be clearly identified (Figures 8, S14). Due to the cell division, the cell density was higher, so most of the cells were in direct contact with each other. Nevertheless, the individual cells labeled with two distinct colors of MemGraft-Cy3 and MemGraft-Cy5 probes could still be clearly identified with relatively small color mixing. Some intracellular dots appeared in a different color (Figures 8-D3, S14) indicating that the cells could internalize membrane proteins of other cells, probably originating from dead cells or exosomes. A better understanding of the transfer of membrane proteins between the cells would require a dedicated study. We also challenged our two-color cell tracking approach in video imaging for 1 h. We could clearly see fast dynamics of cell membranes of both green (MemGraft-Cy3) and red (MemGraft-Cy5) cell populations (Video S2). Although MemGraft-Cy5 showed some photobleaching in contrast to MemGraft-Cy3, both probes allowed tracking of cell migration without any visual impact on their behavior. The latter confirmed negligible phototoxicity of these probes, which is essential for the multicolor long-term cell tracking.

### Cell Barcoding

**Figure 9:**
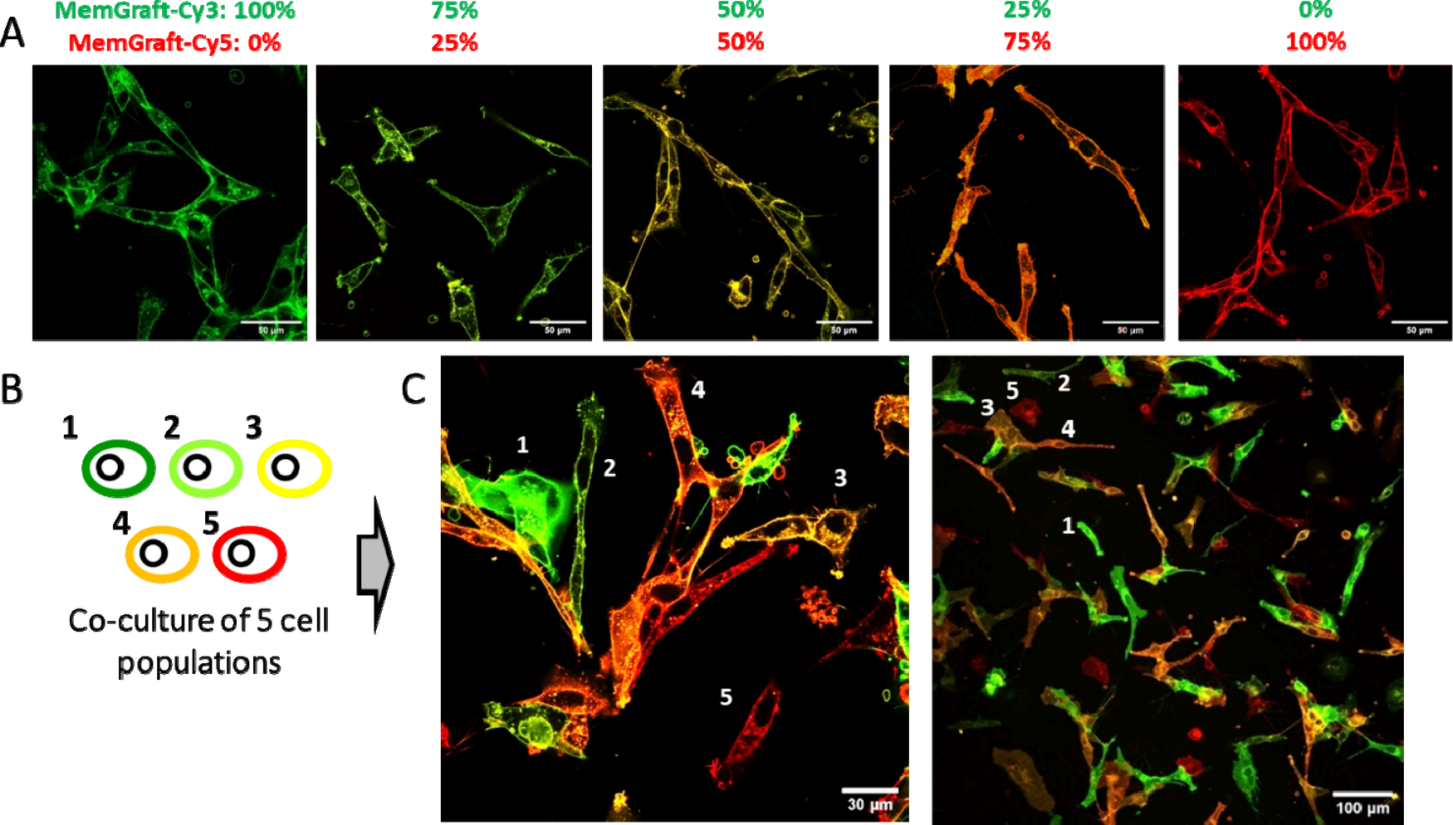
(A) Confocal fluorescence microscopy of U87 cells stained with varied concentration ratio of MemGraft-Cy3/MemGraft-Cy5 (in %): 100/0, 75/25, 50/50, 25/75, 0/100 (left to right: green, lime, yellow, orange, red). Total dye concentration was 1 μM in each case. Scale bar: 50 μm. The images were obtained as a composite of two images for Cy3 (green) and Cy5 (red) channels. (B) Scheme showing that five cell populations where mixed together and cocultured. (C) Confocal fluorescence images (green-red composite) of U87 cells of five barcoded cell populations mixed together and incubated for 3 h before imaging. Total dye concentration was 1 μM in each case. Scale bars: 30 and 100 μm. The numbers indicate cells bearing one of the five barcodes (indicated in panel B).

Inspired by the capacity of MemGraft probes to track cells in two colors without exchange of the Cy3- and Cy5-analogues, we explored the possibility of barcoding the cells in multiple colors using these probes. The covalent labeling of PM could in principle enable costaining of PM with different combinations of green (MemGraft-Cy3) and red (MemGraft-Cy5) probes, thus generating a characteristic barcode for each cell population. To this end, we labeled cells using five probe combinations of MemGraft-Cy3/MemGraft-Cy5 molar %: (1) 100%/0%, (2) 75%/25%, (3) 50%/50%, (4) 25%/75% and (5) 0%/100%. First, individual cell populations labeled with these combinations were imaged using green and red channels, corresponding to Cy3 and Cy5 dyes. Once the images from individual channels were built, the merged (composite) images were constructed by simple combination of the two channels (Figure S15). As a result, we observed that each population was characterized by its own color code, which was homogeneous within the cell population: green, lime, yellow, orange, and red (Figure 9A). This result indicates that the two probes stain cells in a highly homogeneous manner and with a similar efficiency. Then, all five cell populations were detached and then coincubated together for 3 h and imaged by the same method (Figure 9B). We found that the cells displaying different color barcodes were simultaneously present on the surface (Figure 9C). Importantly, all five populations with green, lime, yellow, orange and red barcodes could be readily identified on these images. These observations indicate that after cell detachment of coculture, the barcode was preserved within the individual cells and it did not exchange within different cells. Thus, MemGraft dyes enable barcoding of cells at the level of PM and further tracking of multiple (at least 5) cell populations simultaneously, using just two probes.

### Cell Biotinylation and Manipulation

**Figure 10:**
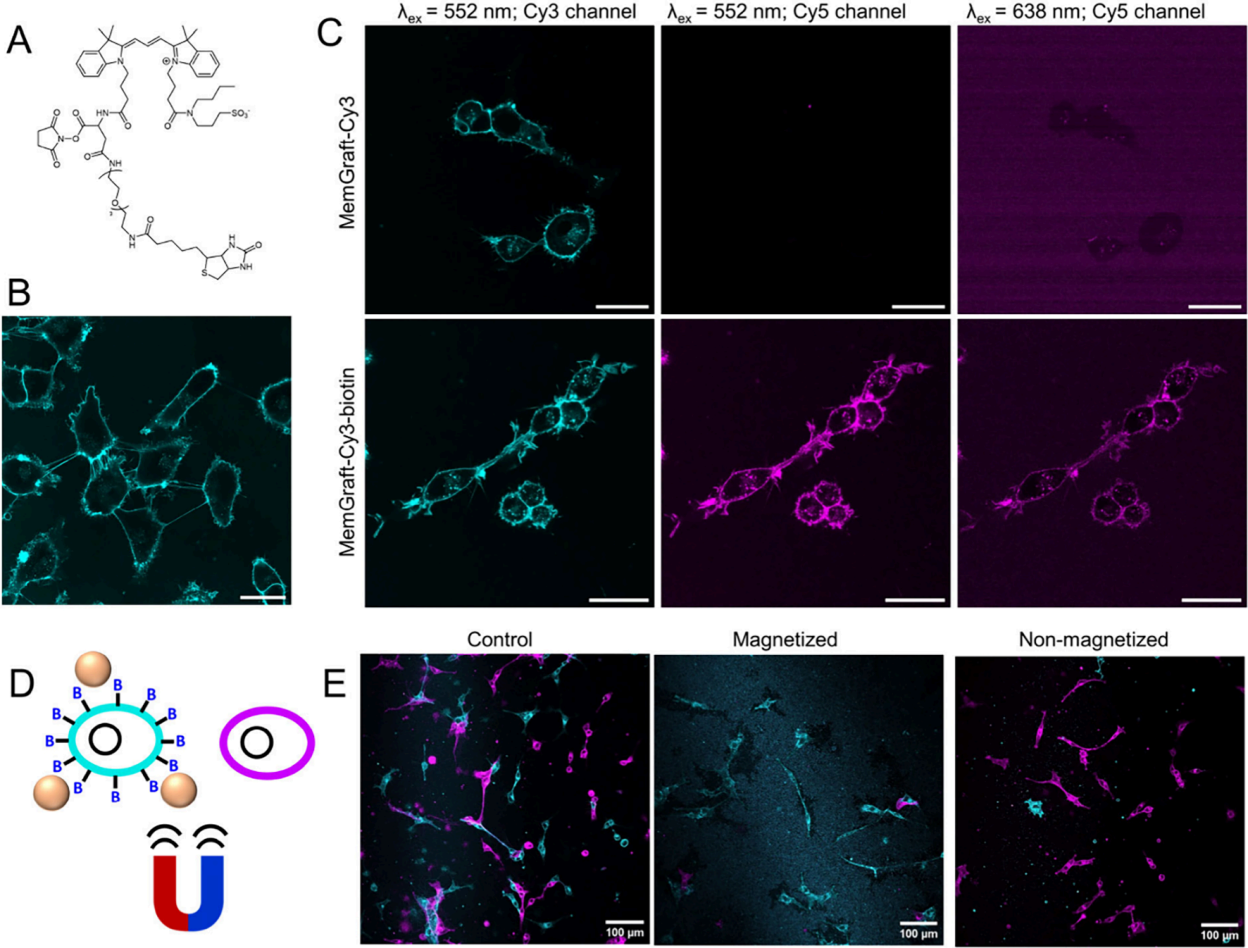
(A) Chemical structure of MemGraft-Cy3-biotin. (B) Confocal fluorescence microscopy of U87 cells stained with MemGraft-Cy3-biotin. Dye concentration was 500 nM. Scale bar: 30 μm. (C) Confocal fluorescence microscopy of U87 cells stained with MemGraft-Cy3 (upper panels) and MemGraft-Cy3-biotin (lower panels) and incubated with the streptavidin-Cy5 conjugate.Left panel: image recorded with excitation of Cy3 (552 nm) and emission of Cy3. Middle panel: the image is recorded with excitation of Cy3 (552 nm) and emission of Cy5. Right panel: the image is recorded with excitation of Cy5 (638 nm) and emission of Cy5. MemGraft probe concentration was 500 nM in each case. The cells were washed and incubated for 30 min with streptavidin-Cy5 (0.1 mg/mL). Scale bar: 30 μm. (D) Scheme of magnetic separation of biotinylated cells (in cyan) using magnetic beads (colored in beige) from nonbiotinylated ones (in magenta). (E) Confocal fluorescence microscopy of U87 cells stained separately with MemGraft-Cy3-biotin (cyan) and MemGraft-Cy5 (magenta) and mixed together. Dye concentration was 500 nM. Left panel: control cells mixed without further treatment. Middle panel: magnetized fraction of cells incubated with streptavidin magnetic beads and isolated using a magnetic stand. Right panel: nonmagnetized fraction of cells from the supernatant after incubation with streptavidin magnetic beads and placing on a magnetic stand. Scale bars: 100 μm.

The capacity of MemGraft probes to label covalently live cells at their plasma membrane opens unique opportunities in cell surface engineering, which is a rapidly developing research direction.^61,62^ To address this point, we designed a MemGraft-Cy3 derivative bearing a biotin residue through a PEG3 linker (MemGraft-Cy3-biotin, Figure 10A). Biotin is an attractive plug-and-play unit, which enables specific conjugation and manipulation using biotin–streptavidin coupling. Incubation of cells with MemGraft-Cy3-biotin resulted in specific plasma membrane labeling, which showed that the modification of MemGraft-Cy3 with PEG3 and biotin units did not alter its PM-specific labeling (Figure 10B). Next, we verified whether the attached biotin unit is capable to interact with streptavidin. To this end, cells labeled with MemGraft-Cy3-biotin were incubated with streptavidin-Cy5 conjugate and then imaged at three channels: (1) the Cy3 channel to visualize MemGraft-Cy3 part; (2) Cy5 emission channel with Cy3 excitation in order to eventually observe Forster Resonance Energy Transfer (FRET) from MemGraft-Cy3 to attached streptavidin-Cy5 (FRET channel) and (3) at the Cy5 emission channel with Cy5 excitation in order to detect streptavidin-Cy5 part (direct Cy5 channel). We found a strong membrane-specific signal at the Cy5 channel with Cy3-excitation and its colocalization with the signal at the Cy3 channel (Figure 10C). Direct excitation of Cy5 also produced membrane-specific signal, but the background noise was much more significant. In the case of control dye MemGraft-Cy3 (without biotin), a much weaker signal was observed at the FRET channel, whereas in the direct Cy5 channel only background noise was observed. Another control experiment with nonlabeled cells treated with strepatvidin-Cy5 showed no signal from cell membranes in any of the studied channels (Figure S16). These observations provide evidence of specific attachment of streptavidin-Cy5 to the cells labeled with MemGraft-Cy3-biotin. The latter leads to the close proximity of the Cy5 label with the MemGraft-Cy3 part, leading to the observed FRET signal. In contrast, a much weaker signal observed for the FRET signal with the control MemGraft-Cy3 corresponds to the leakage of the Cy3 emission into the Cy5 emission channel. It should be noted that direct Cy5 channel also confirmed the attachment of streptavidin-Cy5 conjugate, but the quality of the signal was much worse because, in this case, all streptavidin-Cy5 conjugates were excited, including those bound nonspecifically to the glass surface. Thus, the FRET approach provides a more specific signal of streptavidin-Cy5 fraction bound to the plasma membrane.

Ultimately, we tested whether the MemGraft-based cell-biotinylation approach could be used for cell manipulation. To this end, we applied streptavidin-coated magnetic beads, which are commonly used to separate biotinylated molecules or cells from nonbiotinylated ones. In our experimental setup, one cell population was labeled with MemGraft-Cy3-biotin, while the other one was labeled with nonbiotinylated MemGraft-Cy5 (Figure 10D). Then, the cells were detached and mixed together. This control conditions gave two cell populations, corresponding to biotinylated cells stained in cyan by MemGraft-Cy3-biotin and nonbiotinylated ones stained in magenta by MemGraft-Cy5, as evidenced by two-color fluorescence imaging (Figure 10E). In parallel, the suspension of the two cell populations was exposed to a magnetic field, which resulted in rapid precipitation of some fraction of cells, which was isolated from the supernatant (Figure 10D). Then, the isolated magnetized and remaining (nonmagnetized) supernatant suspensions were deposited on glass slides and imaged. Remarkably, the magnetized fraction showed predominantly cyan color code of the cells, whereas the remaining nonmagnetized suspension was stained in magenta (Figure 10E). These experiments provided the proof of concept of cell manipulation coupled to fluorescence imaging using the MemGraft approach in combination with the biotin–streptavidin coupling strategy. The advantage of using MemGraft strategy is linked to the higher labeling density, as it was evidenced from much more efficient cell labeling by MemGraft compared to the classical NHS labeling approach (see Figures 3 and 4). Moreover, the fluorescence modality of MemGraft allows direct observation of cell surface modification and its possible impact on the plasma membrane morphology by fluorescence microscopy.

## Discussion

Staining cell surface is generally done by membrane probes that target plasma membranes by hydrophobic noncovalent interactions. However, this labeling is reversible and prone to dye exchange, which may lead to a number of limitations caused by dye leakage into a biological medium. Permanent (covalent) labeling of plasma membranes could address these limitations but remains challenging. First, the right molecular target for PM should be chosen for labeling. Natural lipids constituting cell plasma membranes are not suitable for direct chemical modification, unless metabolic labeling is used, which allows introduction of reactive biorthogonal groups.^5^ However, this strategy has limitations because lipids can still exchange with the biological medium containing serum, and they are not compatible with fixation and permeabilization. Robust labeling of cell surface that overcomes limitations of lipids could be achieved by nonspecific labeling of membrane proteins. However, robust techniques for protein labeling, such as protein tag technologies^39−43^ and affinity-based labeling,^45,46^ are designed to target specific proteins and not the whole population present on the cell surface. Nonspecific labeling of cell surface proteins by NHS esters or maleimides is rather inefficient, which was confirmed in the present work using commercially available derivatives. The latter can be explained by relatively slow reaction kinetics of NHS esters amino groups of proteins and poor exposure of SH-groups of membrane proteins outside the cells in the case of maleimides. Therefore, these reagents require high dye concentrations and a rather long reaction time, which leads to off-target labeling. In this work, we propose a concept of the cell surface labeling based on lipid-directed chemical grafting to membrane proteins. We use lipid anchors to favor reversible interactions of the dye with lipid membrane^11,38^ in order to enhance its local concentration in the surrounding of membrane proteins. In suspension of cells at concentration of 10^5^ cells per 1 mL, the total lipid concentration on the outer membrane leaflet of cells can be estimated as ∼0.33 μM (assuming the cell diameter of 20 μm and area per lipid^63^ of 0.6 nm^2^). Then, let us assume that at 100 nM concentration of MemGraft, only 1% of the dye partitions into cell plasma membranes due to its low affinity. Then, the local dye concentration would correspond to ∼0.3 mol % vs lipids, i.e., ∼4 mM vs lipids (assuming average molecular weight of a lipid is 750 g/mol). This high local concentration can explain why labeling of the cell surface is so efficient at low concentrations of MemGraft (100 nM), in contrast to commercial derivatives, unable to label cell membranes at these conditions. Limited reactivity of amino-groups at the biomembrane surface for the commercial water-soluble dye, like disulfo-Cy3-NHS, is also related to the neutral pH (7.4) used for cell labeling, which leads to protonation (i.e., deactivation) of water exposed amino groups. As sulfo-NHS-acetate inhibited cell labeling by disulfo-Cy3-NHS, but not by MemGraft-Cy3, we concluded that MemGraft can assess amino groups shielded from water at the membrane interface. These amino groups are probably not protonated at neutral pH and therefore more reactive than water-exposed amino groups, which provides an additional explanation for the higher reactivity of MemGraft-Cy3 versus commercial disulfo-Cy3-NHS.

The other important challenge in PM labeling is endocytosis,^64,65^ which results in rapid internalization of labeled molecules inside the cells. Endocytosis is typically observed for classical noncovalent membrane probes, which show tendency to accumulate inside endosomes over time.^4,66^ The same should concern probes that label covalently membrane proteins. Our results indeed show that after labeling, the MemGraft signal increases inside the cells in the perinuclear regions corresponding to endosomes (Figure 6). However, it is remarkable to note that even after 29 h of incubation, the major part of fluorescence is still observed on the cell surface (Figure 8). Taking in account that 50% of plasma membrane can be internalized within ∼10 min,^67^ the observed PM labeling after 29 h suggest that the large fraction of membrane proteins labeled by MemGraft was recycled to the cell surface through intrinsic membrane recycling pathways.^68,69^ In this respect, our probes could be a powerful tool to study membrane protein trafficking, recycling, and degradation.

We foresee that the developed strategy will find direct applications in a number of advanced fluorescence microscopy methods. Primarily, the capacity of our probe to work in serum will enable long-term imaging and tracking of cells in native conditions, which is currently done using fluorescent nanoparticles.^70−73^ Here, the main limitations will be the cell division, which will systematically divide by two the labeling level and eventual degradation of the membrane proteins after multiple recycling processes. Second, availability of MemGraft in multiple colors enables cell barcoding and tracking of multiple cell populations. Here, we realized 5 barcoded populations based on two dyes, and we expect that with addition of the third color, it will be possible to make at least 13 color codes. Currently, the closest technique that enable multicolor cell barcoding and imaging is Brainbow,^74,75^ a powerful tool to visualize organization and connections of neurons in brain. However, our approach is simpler and does not require genetic modification. The only direct methods for cell barcoding use combination of fluorescent nanoparticles of different colors,^73,76^ but they label endocytic vesicles and not the cell contour. The covalent nature of labeling opens other multiple opportunities. Primarily, it makes MemGraft compatible with fixation and permeabilization methods, including standard immunostaining. Moreover, we expect that the capacity of MemGraft probes to link covalently to membrane proteins could address the problem of lateral diffusion of membrane probes in single-molecule localization microscopy,^34−38^ and thus allow imaging cell membranes with superior resolution. Ultimately, we expect that MemGraft approach with appropriate fluorophores will enable plasma membrane studies by advanced imaging methods such as expansion microscopy,^26−28^ tissue/animal clearing^29−31^ and correlative light and electron microscopy (CLEM),^32,33^ all requiring covalent labeling.

Biotinylated MemGraft introduces additional functionality to our grafting strategy beyond imaging. On the one hand, nonspecific labeling of membrane proteins with biotin tag would enable proteomics analysis,^77−79^ for example, to understand membrane protein trafficking. On the other hand, our data on cell biotinylation by MemGraft strategy opens the perspective of cell surface engineering,^61,80^ which enables functionalization of cell surface with synthetic molecules^80,81^ and biomolecules,^82−84^ for controlling and studying cell adhesion, cell–cell interactions, cell manipulation and delivery. We expect that the lipid-driven covalent grafting strategy could facilitate introduction of functional units to the surface of live cells, opening countless number of opportunities in this rapidly expanding field.

## Conclusion

Permanent labeling of cell plasma membranes remains a challenge because of the noncovalent nature of current fluorescent membrane probes. Here, we report the concept of lipid-directed covalent labeling of plasma membranes. We propose to exploit transient binding to the biomembrane surface as a driving force creating a high local concentration of the dye in proximity of membrane proteins, which can catalyze ligation to membrane proteins. We designed a new family of dyes, named MemGraft, based on a cyanine dye that bears a low-affinity membrane anchor and an activated ester (or maleimide) on two ends of the dye. We found that the low-affinity anchor plays a crucial role in ensuring efficient PM labeling, as a control compound without this group failed to stain PM. Moreover, MemGraft probes with activated ester and maleimide were found to be superior to corresponding commercial NHS-ester and maleimide Cy3-based labels. These data confirmed that the anchor ensures transient binding to the plasma membrane that accelerates the labeling of amino or thiol groups of membrane proteins in the proximity of lipid membranes. The reactivity of the NHS-ester was found to be crucial, as less reactive fluorophenyl activated esters showed strong internalization and inefficient PM labeling. SDS-page confirmed that multiple membrane proteins were labeled with MemGraft-Cy3 probes (with activated ester and maleimide), thus confirming that our strategy of lipid-driven covalent labeling of the cell surface operated through the nonselective conjugation to the membrane proteins. In comparison to conventional noncovalent staining of PM by reference probes (e.g., MemBright), the MemGraft approach revealed several critical advantages: resistance to full culture medium with serum in long-term cellular experiments and compatibility with fixation and permeabilization. Additionally, these probes enabled long-term labeling of cells and coculture of two cell populations stained in different colors without dye exchange, which was not possible before with conventional membrane probes. The latter allowed for imaging of cell–cell interactions and visualization of cell–cell contacts. Moreover, a combination of different ratios of MemGraft-Cy3 and MemGraft-Cy5 probes (NHS-esters) enabled long-term cell barcoding in at least 5 color codes, which opens new opportunities in tracking and visualizing multiple cell populations, similar to those offered by “Brainbow” method, but without genetic modification of the cells. Ultimately, based on the MemGraft strategy, we designed a fluorescent probe for efficient biotinylation of the cell surface, which enabled cell manipulation using streptavidin-coated magnetic beads. The developed MemGraft concept opens a new chapter in the design of advanced PM probes and, in a more general sense, proposes an efficient approach for the covalent functionalization of any biomembrane surface and cell surface engineering.

## Supporting Information Available

Part of Materials and Methods section, including protocols of synthesis of all dyes, cytotoxicity, model membrane conjugation, protein extraction and quantification assays, and SDS-PAGE analysis; additional experimental data, including cellular imaging, NMR and mass analysis, SDS-PAGE and cytotoxicity evaluation (PDF)
Video S1. Fluorescence video imaging of U87 cells labelled with MemGraft-Cy3 (MP4)
Video S2. Fluorescence video imaging of co-seeded U87 cells labelled with MemGraft-Cy3 (green) and MemGraft-Cy5 (red) (MP4)
