# Supplementary material for: Lipid-Directed Covalent Labeling of Plasma Membranes for Long-Term Imaging, Barcoding and Manipulation of Cells
Source: JACS Au. 2025 Feb 12;5(2):922–36. doi: 10.1021/jacsau.4c01134 (PMC11863151; doi:10.1021/jacsau.4c01134)
Supplement: Supplementary file 1 — au4c01134_si_001.pdf [file au4c01134_si_001.pdf]

Supporting information

Lipid-directed covalent labeling of plasma membranes for long-term imaging, barcoding and manipulation of cells

Nathan Aknine, Remi Pelletier and Andrey S. Klymchenko*

Laboratoire de Bioimagerie et Pathologies, UMR 7021 CNRS, ITI SysChem, Faculté de Pharmacie, Université de Strasbourg, 67401 Illkirch, France.

E-mail: andrey.klymchenko@unistra.fr

Materials and methods

Synthesis

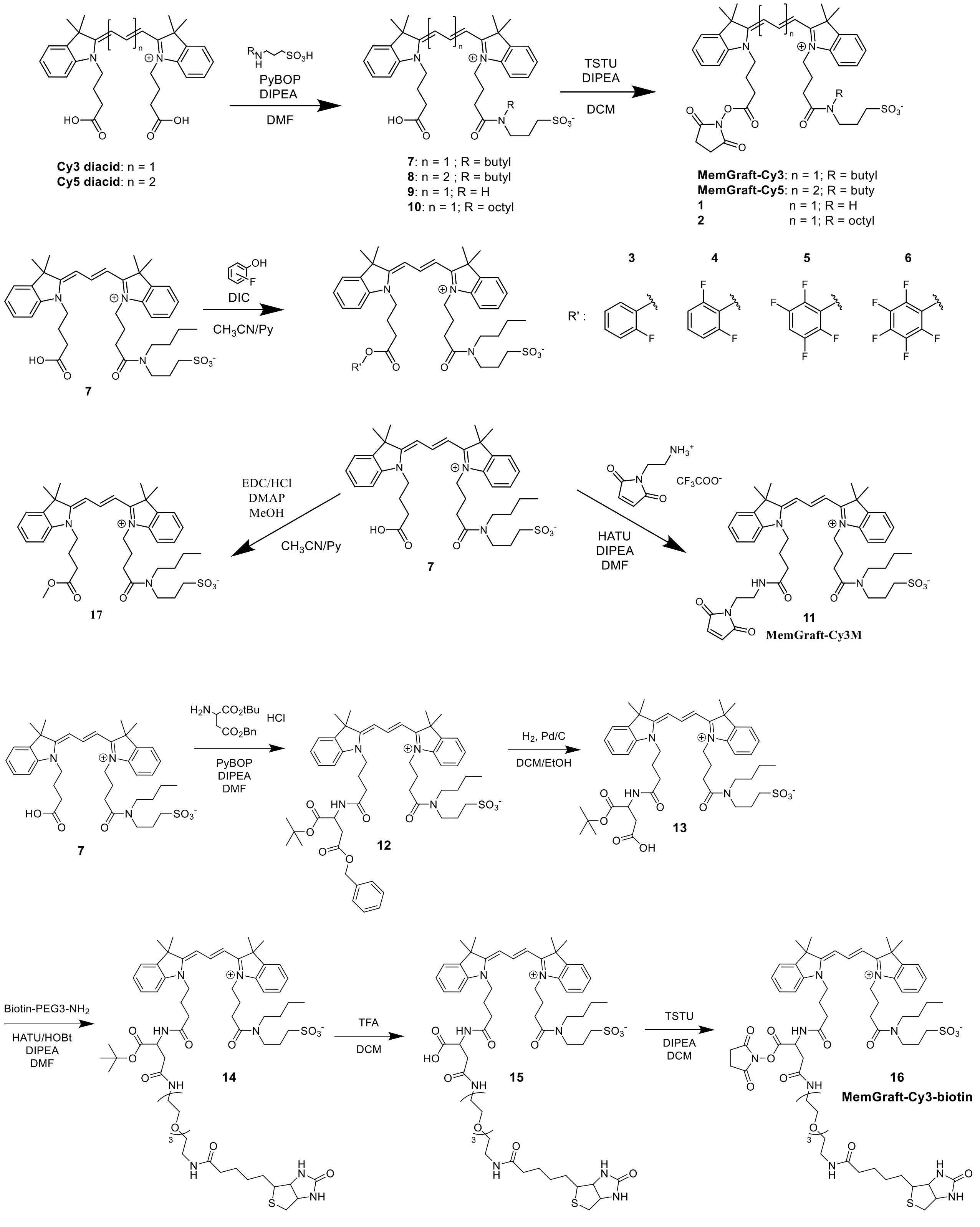

Figure S1. Synthesis scheme for cyanine based MemGraft probes.

3-(N-butyl-4-(2-((E)-3-((Z)-1-(3-carboxypropyl)-3,3-dimethylindolin-2-ylidene)prop-1-en-1-yl)-3,3-dimethyl-3H-indol-1-ium-1-yl)butanamido)propane-1-sulfonate (7).

The synthesis is adapted from the protocol decreased elsewhere.[1] Compound Cy3 diacid chloride (110 mg, 204.8 µmol) was dissolved in dry DMF together with 3-(butylammonio)propane-1-sulfonate (40.0 mg, 204.8 µmol) and DIPEA (125 µL, 716.9 µmol). The mixture was cooled to 0°C and PyBOP (106.6 mg, 204.8 µmol) was added. The mixture was allowed to stir at room temperature for 4h (controlled by TLC). The reaction was quenched with water and the mixture was evaporated *in vacuo*. The crude product was purified by flash chromatography on silica SiO_2 gel with gradual eluting with DCM/methanol from 98:2 to 85:15 v/v %. Yield: 50 mg (36%) as a dark magenta solid. ^{1}H NMR (500 MHz, MeOD) δ ppm: 8.43 (td, *J* = 13.4, 6.6 Hz, 1H), 7.46 – 7.41 (m, 2H), 7.38 – 7.30 (m, 4H), 7.20 (tt, *J* = 7.6, 2.6 Hz, 2H), 6.55 – 6.41 (m, 2H), 4.12 (td, *J* = 10.5, 6.5 Hz, 4H), 3.45 (tq, *J* = 12.0, 5.4 Hz, 2H), 3.31 – 3.18 (m, 4H), 2.51 – 2.43 (m, 2H), 2.00 (dd, *J* = 41.2, 22.8, 9.7, 6.7 Hz, 7H), 1.66 (dd, *J* = 3.9, 1.4 Hz, 12H), 1.42 (tt, *J* = 8.8, 6.4 Hz, 2H), 1.23 – 1.12 (m, 4H), 0.82 (dd, *J* = 15.2, 7.6 Hz, 3H). ^{13}C NMR (126 MHz, MeOD) δ ppm: 174.72, 172.43, 150.95, 150.77, 141.96, 140.82, 128.66, 128.27, 125.37, 125.03, 122.14, 119.07, 111.16, 109.00, 102.68, 49.26, 48.53, 46.55, 45.65, 44.68, 43.31, 42.44, 30.97, 30.36, 29.34, 28.67, 28.24, 26.98, 24.22, 23.91, 23.01, 22.52, 22.23, 12.87. HRMS (ESI), m/z: [M+H]+ calcd for $C_{38}H_{52}N_3O_6S^+$, 678.3571; found, 678.3585.

3-(N-butyl-4-(2-((1E,3E)-5-((Z)-1-(3-carboxypropyl)-3,3-dimethylindolin-2-ylidene)penta-1,3-dien-1-yl)-3,3-dimethyl-3H-indol-1-ium-1-yl)butanamido)propane-1-sulfonate (8).

The synthesis is adapted from the protocol decreased elsewhere.[1] Compound Cy5 diacid chloride (98 mg, 174.0 µmol) was dissolved in dry DMF together with 3-(butylammonio)propane-1-sulfonate (34.5 mg, 174.0 µmol) and DIPEA (106 µL, 609.1 µmol). The mixture was cooled to 0°C and PyBOP (90.6 mg, 174.0 µmol) was added. The mixture was allowed to stir at room temperature for 4h (controlled by TLC). The reaction was quenched with water and the mixture was evaporated *in vacuo*. The crude product was purified by flash chromatography on silica SiO_2 gel with gradual eluting with DCM/methanol from 98:2 to 85:15 v/v %. Yield: 17.5 mg (14%) as a dark blue solid. ^{1}H NMR (400 MHz, MeOD) δ ppm: 8.12 (t, *J* = 12.9 Hz, 2H), 7.37 (dt, *J* = 7.2, 2.6 Hz, 2H), 7.33 – 7.20 (m, 4H), 7.13 (dtt, *J* = 9.1, 3.5, 1.3 Hz, 2H), 6.58 (dt, *J* = 23.6, 12.3 Hz, 1H), 6.46 – 6.17 (m, 2H), 4.08 (q, *J* = 10.8 Hz, 4H), 3.45 – 3.38 (m, 2H), 2.79 – 2.72 (m, 2H), 2.41 (dt, *J* = 21.7, 6.4 Hz, 3H), 1.97 (q, *J* = 8.1 Hz, 6H), 1.60 (d, *J* = 2.3 Hz, 12H), 1.41 (dd, *J* = 10.0, 5.4 Hz, 2H), 1.29 – 1.12 (m, 6H), 0.84 – 0.78 (m, 3H). ^{13}C NMR (101 MHz, MeOD) δ ppm: 172.06, 171.69, 170.93, 170.80, 152.80, 152.48, 140.63, 139.85, 139.75, 139.68, 126.90, 124.19, 123.51, 123.31, 120.50, 109.26, 109.11, 101.51, 52.97, 47.66, 47.24, 46.96, 45.00, 44.11, 41.43, 40.92, 29.03, 27.74, 27.03, 26.71, 25.05, 22.68, 21.72, 21.10, 20.90, 20.67, 18.36, 18.23, 11.36, 10.27. HRMS (ESI), m/z: [M+H]+ calcd for $C_{40}H_{53}N_3O_6S$, 703.3655; found, 703.3642.

3-(4-(2-((E)-3-((Z)-1-(4-((2,5-dioxopyrrolidin-1-yl)oxy)-4-oxobutyl)-3,3-dimethylindolin-2-ylidene)prop-1-en-1-yl)-3,3-dimethyl-3H-indol-1-ium-1-yl)butanamido)propane-1-sulfonate (9).

Compound Cy3 diacid (40 mg, 68.7 µmol) was dissolved in dry DMF together with 3-aminopropane-1-sulfonic acid (10.5 mg, 76.0 µmol) and DIPEA (43 µL, 246.8 µmol). The mixture was cooled to 0°C

and PyBOP (40 mg, 76.0 µmol) was added. The mixture was allowed to stir at room temperature for 4h (controlled by TLC). The reaction was quenched with water and the mixture was evaporated *in vacuo*. The crude product was purified by preparative TLC (SiO_2, DCM/MeOH, 85:15 v/v %). Yield: 15 mg (35%) as a dark magenta solid. ^{1}H NMR (400 MHz, MeOD) δ ppm: 8.61 – 8.51 (m, 1H), 7.61 – 7.52 (m, 2H), 7.50 – 7.38 (m, 4H), 7.38 – 7.26 (m, 2H), 6.71 – 6.55 (m, 2H), 4.22 (p, *J* = 5.9 Hz, 4H), 3.35 (s, 4H), 2.90 (dt, *J* = 29.0, 7.3 Hz, 2H), 2.64 – 2.47 (m, 4H), 2.13 (pd, *J* = 9.7, 5.0 Hz, 6H), 2.03 – 1.84 (m, 2H), 1.77 (d, *J* = 1.7 Hz, 12H). ^{13}C NMR (101 MHz, MeOD) δ 174.72, 173.46, 173.00, 150.94, 141.93, 140.82, 128.63, 128.10, 125.38, 122.12, 111.10, 102.56, 54.68, 50.90, 49.27, 48.94, 43.30, 43.07, 38.10, 31.75, 29.96, 28.17, 26.93, 24.81, 22.75, 22.00, 13.03. HRMS (ESI), m/z: [M+H]+ calcd for $C_{34}H_{43}N_3O_6S$, 621.2873; found, 621.2879.

3-(4-(2-((E)-3-((Z)-1-(3-carboxypropyl)-3,3-dimethylindolin-2-ylidene)prop-1-en-1-yl)-3,3-dimethyl-3H-indol-1-ium-1-yl)-N-octylbutanamido)propane-1-sulfonate (10)

Compound Cy3 diacid chloride (110 mg, 204.8 µmol) was dissolved in dry DMF together with 3-(octylammonio)propane-1-sulfonate (40.0 mg, 204.8 µmol) and DIPEA (125 µL, 716.9 µmol). The mixture was cooled to 0°C and PyBOP (106.6 mg, 204.8 µmol) was added. The mixture was allowed to stir at room temperature for 4h (controlled by TLC). The reaction was quenched with water and the mixture was evaporated *in vacuo*. The crude product was purified by flash chromatography on silica SiO_2 gel with gradual eluting with DCM/methanol from 98:2 to 85:15 v/v %. Yield: 51 mg (36%) as a dark magenta solid. ^{1}H NMR (400 MHz, $CDCl_3$) δ 8.45 – 8.31 (m, 1H), 7.39 (q, *J* = 6.8 Hz, 3H), 7.32 (dd, *J* = 7.4, 2.5 Hz, 3H), 7.22 (tdd, *J* = 8.8, 6.4, 3.9 Hz, 2H), 6.97 – 6.77 (m, 2H), 4.22 (s, 2H), 3.64 – 3.49 (m, 2H), 3.29 (d, *J* = 7.6 Hz, 2H), 3.19 (s, 4H), 3.16 – 3.05 (m, 4H), 2.93 (d, *J* = 8.7 Hz, 4H), 2.81 (t, *J* = 7.0 Hz, 2H), 2.76 – 2.62 (m, 2H), 2.42 – 2.31 (m, 4H), 2.18 – 2.00 (m, 6H), 1.69 (s, 12H), 1.35 – 1.10 (m, 10H), 0.82 (s, 3H). ^{13}C NMR (101 MHz, $CDCl_3$) δ 173.67, 172.01, 150.79, 141.93, 141.90, 140.43, 140.40, 129.19, 129.13, 125.43, 125.30, 121.92, 121.81, 111.84, 111.38, 103.99, 54.11, 49.13, 48.97, 48.78, 48.17, 47.41, 47.22, 46.69, 45.92, 44.20, 42.35, 31.80, 31.72, 30.10, 29.69, 29.46, 29.42, 29.27, 29.23, 29.16, 29.10, 28.25, 28.16, 28.12, 27.93, 27.08, 26.93, 26.74, 25.97, 24.92, 23.54, 22.62, 22.59, 21.85, 19.03, 14.07, 14.06. HRMS (ESI), m/z: [M+H]+ calcd for $C_{42}H_{59}N_3O_6S$, 733.4125; found, 733.4134.

3-(N-butyl-4-(2-((E)-3-((Z)-1-(4-(2-fluorophenoxy)-4-oxobutyl)-3,3-dimethylindolin-2-ylidene)prop-1-en-1-yl)-3,3-dimethyl-3H-indol-1-ium-1-yl)butanamido)propane-1-sulfonate (3).

Compound **7** (20 mg, 29.5 µmol) was added to a stirred solution of diisopropylcarbodiimide (DIC) (5 µL, 35.4 µmol) in CH_3CN/Pyridine (9:1). The mixture was allowed to stir for 15 minutes. 2-fluorophenol (3.5 µL, 35.4 µmol) was slowly added and the reaction was stirred for 12h. The solvent was evaporated *in vacuo* and the crude product was purified by flash chromatography on silica SiO_2 gel with gradual eluting with DCM/methanol from 98:2 to 90:10 v/v %. Yield: 9 mg (40%) as a dark magenta solid. ^{1}H NMR (500 MHz, $CDCl_3$) δ ppm: 8.37 (t, *J* = 13.4 Hz, 1H), 7.33 – 7.24 (m, 6H), 7.18 – 7.06 (m, 8H), 4.44 – 4.37 (m, 2H), 4.16 (t, *J* = 7.6 Hz, 2H), 3.69 (dd, *J* = 9.9, 6.6 Hz, 2H), 3.26 (t, *J* = 7.5 Hz, 2H), 3.12 (t, *J* = 6.4 Hz, 2H), 2.94 – 2.89 (m, 4H), 2.23 – 2.11 (m, 4H), 1.65 (d, *J* = 4.5 Hz, 12H), 1.42 (ddt, *J* = 9.2, 7.7, 3.5 Hz, 2H), 1.27 – 1.16 (m, 4H), 0.83 (t, *J* = 7.3 Hz, 3H). ^{13}C NMR (126 MHz, $CDCl_3$)

δ ppm: 174.00, 173.85, 172.52, 171.68, 151.48, 142.16, 141.91, 140.59, 140.45, 138.09, 129.09, 128.94, 127.03, 125.19, 124.51, 123.92, 121.86, 116.68, 116.53, 111.24, 105.10, 104.53, 48.96, 47.79, 46.91, 44.34, 43.50, 36.46, 33.95, 31.42, 31.03, 30.10, 28.19, 25.81, 25.66, 24.98, 24.63, 20.18, 13.88. HRMS (ESI), m/z: [M+H]+ calcd for $C_{44}H_{54}FN_3O_6S$, 771.3717; found, 771.3706.

3-(N-butyl-4-(2-((E)-3-((Z)-1-(4-(2,6-difluorophenoxy)-4-oxobutyl)-3,3-dimethylindolin-2-ylidene)prop-1-en-1-yl)-3,3-dimethyl-3H-indol-1-ium-1-yl)butanamido)propane-1-sulfonate (4).

Compound **7** (21 mg, 30.9 µmol) was added to a stirred solution of DIC (6 µL, 37 µmol) in CH_3CN/Pyridine (9:1). The mixture was allowed to stir for 15 minutes. 2,4-difluorophenol (3.5 µL, 37 µmol) was slowly added and the reaction was stirred for 12h. The solvent was evaporated *in vacuo* and the crude product was purified by preparative TLC (SiO_2, DCM/MeOH, 85:15 v/v%). Yield: 20 mg (82%) as a dark magenta solid. ^1H NMR (400 MHz, $CDCl_3$) δ ppm: 8.43 (t, *J* = 13.4 Hz, 1H), 7.44 – 7.27 (m, 6H), 7.25 – 7.08 (m, 5H), 6.96 – 6.80 (m, 2H), 4.50 – 4.41 (m, 2H), 4.22 (t, *J* = 7.6 Hz, 2H), 3.76 (dd, *J* = 10.0, 6.4 Hz, 2H), 3.32 (t, *J* = 7.5 Hz, 2H), 3.20 (t, *J* = 6.4 Hz, 2H), 3.02 – 2.95 (m, 2H), 2.85 (t, *J* = 8.0 Hz, 2H), 2.21 (dd, *J* = 23.6, 6.7 Hz, 4H), 1.71 (d, *J* = 3.6 Hz, 12H), 1.49 (tt, *J* = 8.0, 6.4 Hz, 3H), 1.29 – 1.24 (m, 4H), 0.89 (t, *J* = 7.3 Hz, 3H). ^{13}C NMR (101 MHz, $CDCl_3$) δ ppm: 173.93, 172.56, 171.78, 151.51, 142.16, 141.91, 140.60, 140.46, 128.96, 125.21, 124.47, 121.88, 105.26, 105.05, 104.77, 104.58, 48.95, 30.16, 29.97, 29.70, 28.21, 25.82, 24.68, 22.30, 20.19, 13.89. HRMS (ESI), m/z: [M+H]+ calcd for $C_{44}H_{53}F_2N_3O_6S$, 789.3623; found, 789.3644.

3-(N-butyl-4-(2-((E)-3-((Z)-3,3-dimethyl-1-(4-oxo-4-(2,3,5,6-tetrafluorophenoxy)butyl)indolin-2-ylidene)prop-1-en-1-yl)-3,3-dimethyl-3H-indol-1-ium-1-yl)butanamido)propane-1-sulfonate (5)

Compound **7** (24 mg, 35.4 µmol) was added to a stirred solution of DIC (6.6 µL, 43 µmol) in CH_3CN/Pyridine (9:1). The mixture was allowed to stir for 15 minutes. 2,3,5,6-tetrafluorophenol (7 mg, 43 µmol) was slowly added and the reaction was stirred for 12h. The solvent was evaporated *in vacuo* and the crude product was purified by preparative TLC (SiO_2, DCM/MeOH, 85:15 v/v%). Yield: 15 mg (52%) as a dark magenta solid. ^1H NMR (400 MHz, $CDCl_3$) δ ppm: 8.48 – 8.34 (m, 1H), 7.49 – 7.26 (m, 6H), 7.24 – 7.07 (m, 3H), 6.98 (tt, *J* = 9.9, 7.1 Hz, 1H), 6.44 (tt, *J* = 10.2, 6.9 Hz, 1H), 4.47 – 4.38 (m, 2H), 4.21 (t, *J* = 7.7 Hz, 2H), 3.84 (p, *J* = 6.5 Hz, 2H), 3.75 (q, *J* = 7.4 Hz, 2H), 3.34 (q, *J* = 7.3 Hz, 2H), 3.27 (t, *J* = 6.6 Hz, 2H), 3.04 – 2.94 (m, 2H), 2.89 – 2.78 (m, 2H), 2.33 – 2.23 (m, 2H), 2.23 – 2.08 (m, 4H), 1.71 (d, *J* = 2.3 Hz, 12H), 1.49 (p, *J* = 7.7 Hz, 2H), 1.36 – 1.17 (m, 6H), 0.90 (q, *J* = 7.1 Hz, 3H). ^{13}C NMR (101 MHz, $CDCl_3$) δ ppm: 174.08, 173.95, 172.78, 170.18, 157.22, 151.54, 142.06, 141.80, 140.56, 129.04, 125.29, 121.92, 104.85, 104.52, 104.21, 103.11, 102.88, 94.94, 94.71, 94.48, 53.43, 49.24, 48.98, 47.75, 46.94, 45.81, 44.32, 43.29, 42.18, 30.94, 30.10, 29.79, 25.66, 24.60, 23.46, 22.22, 20.19, 14.11, 13.87. HRMS (ESI), m/z: [M+H]+ calcd for $C_{44}H_{51}F_4N_3O_6S$, 825.3435; found, 825.3438.

3-(N-butyl-4-(2-((E)-3-((Z)-3,3-dimethyl-1-(4-oxo-4-(perfluorophenoxy)butyl)indolin-2-ylidene)prop-1-en-1-yl)-3,3-dimethyl-3H-indol-1-ium-1-yl)butanamido)propane-1-sulfonate (6).

Compound **7** (22 mg, 32.5 µmol) was added to a stirred solution of DIC (6 µL, 39 µmol) in CH_3CN/Pyridine (9:1). The mixture was allowed to stir for 15 minutes. 2,3,4,5,6-pentafluorophenol

(6.5 mg, 39 µmol) was slowly added and the reaction was stirred for 12h. The solvent was evaporated *in vacuo* and the crude product was purified by preparative TLC (SiO_2, DCM/MeOH, 85:15 v/v %). Yield: 18 mg (67%) as a dark magenta solid. ^{1}H NMR (400 MHz, $CDCl_3$) δ ppm: 8.49 – 8.33 (m, 1H), 7.35 (tdd, *J* = 14.8, 12.6, 7.3 Hz, 5H), 7.25 – 6.94 (m, 4H), 6.77 (dd, *J* = 30.8, 13.4 Hz, 1H), 4.30 (s, 2H), 4.20 (t, *J* = 7.8 Hz, 2H), 3.84 (p, *J* = 6.4 Hz, 3H), 3.77 – 3.67 (m, 2H), 3.34 (q, *J* = 7.7 Hz, 2H), 2.99 (dd, *J* = 7.5, 3.7 Hz, 2H), 2.82 (dd, *J* = 14.6, 8.3 Hz, 3H), 2.69 (t, *J* = 6.9 Hz, 1H), 2.20 – 2.12 (m, 4H), 1.71 (t, *J* = 1.6 Hz, 12H), 1.30 – 1.23 (m, 4H), 0.90 (td, *J* = 7.4, 5.6 Hz, 3H). ^{13}C NMR (101 MHz, $CDCl_3$) δ ppm: 174.32, 174.15, 173.98, 173.75, 172.87, 170.17, 151.51, 141.99, 141.72, 140.55, 129.07, 125.66, 121.84, 111.71, 111.26, 111.14, 104.67, 104.36, 103.84, 49.27, 47.71, 46.94, 46.69, 44.26, 43.22, 42.25, 30.86, 29.69, 28.14, 25.57, 24.58, 23.89, 23.44, 13.86. HRMS (ESI), m/z: [M+H]+ calcd for $C_{44}H_{50}F_5N_3O_6S$, 843.334; found, 843.3339.

3-(N-butyl-4-(2-((E)-3-((Z)-1-(4-((2-(2,5-dioxo-2,5-dihydro-1H-pyrrol-1-yl)ethyl)amino)-4-oxobutyl)-3,3-dimethylindolin-2-ylidene)prop-1-en-1-yl)-3,3-dimethyl-3H-indol-1-ium-1-yl)butanamido)propane-1-sulfonate (11, MemGraft-Cy3M)

Compound **7** (25 mg, 36.9 µmol) was dissolved in DMF together with N-(2-aminoethyl)maleimide trifluoroacetate salt (10.5 mg, 42 µmol) and DIPEA (23 µL, 132 µmol). The mixture was cooled to 0°C and HATU (16 mg, 42 µmol) was added. The mixture was allowed to stir at room temperature for 4h (controlled by TLC). The reaction was quenched with water and the mixture was evaporated *in vacuo*. The crude product was purified by flash chromatography on silica SiO_2 gel with gradual eluting with DCM/methanol from 98:2 to 85:15 v:v%. Yield: 24 mg (81%) as a dark red solid. ^{1}H NMR (400 MHz, $CDCl_3$) δ 8.39 – 8.26 (m, 2H), 7.24 (m, 7H), 6.94 (d, *J* = 13.4 Hz, 1H), 6.80 (d, *J* = 13.3 Hz, 1H), 6.60 (s, 2H), 4.13 (dt, *J* = 14.5, 7.4 Hz, 4H), 3.63 (t, *J* = 5.7 Hz, 4H), 3.39 (t, *J* = 5.7 Hz, 2H), 3.26 (t, *J* = 7.6 Hz, 2H), 2.85 (t, *J* = 5.6 Hz, 2H), 2.76 (t, *J* = 7.7 Hz, 2H), 2.57 (q, *J* = 5.2 Hz, 2H), 1.64 (s, *J* = 2.6 Hz, 12H), 1.43 (p, *J* = 7.8 Hz, 2H), 1.29 – 1.14 (m, 6H), 0.84 (t, *J* = 7.3 Hz, 3H). ^{13}C NMR (126 MHz, $CDCl_3$) δ 174.15, 173.67, 170.91 (d, *J* = 2.7 Hz), 151.15, 150.36, 141.82, 140.49 (d, *J* = 3.1 Hz), 140.34, 134.11, 129.10 (d, *J* = 15.0 Hz), 125.49, 125.26, 121.86 (d, *J* = 10.7 Hz), 111.79, 111.31, 104.62, 103.85, 53.43, 49.04 (d, *J* = 11.4 Hz), 47.75, 46.95, 44.03 (d, *J* = 12.9 Hz), 38.22, 38.05, 37.60 (d, *J* = 10.7 Hz), 33.12, 32.91, 31.92, 30.92, 30.51 (d, *J* = 13.0 Hz), 30.05 (d, *J* = 3.0 Hz), 29.69, 29.27, 28.21, 28.10, 25.30, 24.30 (d, *J* = 21.0 Hz), 20.19, 13.87.

HRMS (ESI), m/z: [M+H]+ calcd for $C_{44}H_{57}N_5O_7S$, 799.3979; found, 799.3986.

3-(4-(2-((E)-3-((Z)-1-(4-((4-(benzyloxy)-1-(tert-butoxy)-1,4-dioxobutan-2-yl)amino)-4-oxobutyl)-3,3-dimethylindolin-2-ylidene)prop-1-en-1-yl)-3,3-dimethyl-3H-indol-1-ium-1-yl)-N-butylbutanamido)propane-1-sulfonate (12)

Compound **7** (80 mg, 118 µmol) was dissolved in DMF together with H-Asp(OBzl)-OtBu hydrochloride salt (41 mg, 130 µmol) and DIPEA (72 µL, 412 mmol). The mixture was cooled to 0°C and PyBOP (68 mg, 130 µmol) was added. The mixture was allowed to stir at room temperature for 4h (controlled by TLC). The reaction was quenched with water and the mixture was evaporated *in vacuo*. The crude product was purified by flash chromatography on silica SiO_2 gel with gradual eluting with DCM/methanol from 98:2 to 90:10 v:v%. Yield: 75 mg (68%) as a dark red solid. ^{1}H NMR (400 MHz,

$CDCl_3$) δ 8.37 (t, *J* = 13.4 Hz, 1H), 8.11 (d, *J* = 7.7 Hz, 1H), 7.80 (d, *J* = 8.3 Hz, 0H), 7.68 (d, *J* = 8.2 Hz, 0H), 7.41 – 7.16 (m, 17H), 7.00 (d, *J* = 13.4 Hz, 1H), 6.85 (d, *J* = 13.3 Hz, 1H), 6.59 (t, *J* = 12.5 Hz, 0H), 5.11 (dd, *J* = 11.2, 4.7 Hz, 2H), 4.77 (dt, *J* = 7.7, 6.1 Hz, 1H), 4.28 (dd, *J* = 10.5, 6.2 Hz, 2H), 4.20 – 4.09 (m, 3H), 3.69 – 3.58 (m, 2H), 3.28 (t, *J* = 7.5 Hz, 2H), 2.96 – 2.82 (m, 5H), 2.82 – 2.60 (m, 4H), 2.11 (q, *J* = 11.6 Hz, 8H), 1.68 (d, *J* = 4.4 Hz, 14H), 1.38 (s, 9H), 1.26 (dq, *J* = 14.3, 7.1 Hz, 2H), 0.87 (t, *J* = 7.3 Hz, 3H). ^{13}C NMR (101 MHz, $CDCl_3$) δ 174.01, 173.55, 172.97, 172.39, 170.49, 169.79, 151.05, 141.89, 141.86, 140.53, 140.35, 135.79, 129.06, 128.98, 128.47, 128.38, 128.30, 128.12, 125.38, 125.18, 121.92, 121.80, 111.77, 111.24, 104.63, 103.84, 81.90, 66.57, 49.79, 49.06, 48.96, 47.81, 45.66, 44.09, 43.67, 36.57, 32.73, 30.67, 30.07, 28.19, 28.06, 27.86, 27.80, 25.34, 24.27, 23.83, 20.19, 13.89. HRMS (ESI), m/z: [M+H]+ calcd for $C_{53}H_{70}N_4O_9S$, 938.4864; found, 938.4831.

3-(4-(2-((E)-3-((Z)-1-(4-((1-(tert-butoxy)-3-carboxy-1-oxopropan-2-yl)amino)-4-oxobutyl)-3,3-dimethylindolin-2-ylidene)prop-1-en-1-yl)-3,3-dimethyl-3H-indol-1-ium-1-yl)-N-butylbutanamido)propane-1-sulfonate (13)

Compound **12** (35 mg, 37 µmol) was dissolved in a mixture of DCM/EtOH (50:50) and Pd/C (2.7 mg, 2.6 µmol) wad added. The mixture stirred under H_2 at RT for 6h, filtered under Celite; and evaporated *in vacuo*. The crude product was used in the next step without further purification Yield: 31 mg (96%) as a dark red solid. ^{1}H NMR (400 MHz, MeOD) δ 8.45 (t, *J* = 13.4 Hz, 1H), 7.43 (d, *J* = 7.3 Hz, 2H), 7.34 (dd, *J* = 11.9, 3.8 Hz, 4H), 7.20 (dtd, *J* = 8.5, 5.0, 2.1 Hz, 2H), 6.55 – 6.39 (m, 2H), 4.56 (td, *J* = 6.0, 3.3 Hz, 1H), 4.12 (dq, *J* = 12.1, 5.6 Hz, 4H), 3.55 – 3.43 (m, 2H), 3.29 – 3.21 (m, 4H), 2.79 – 2.68 (m, 2H), 2.63 – 2.57 (m, 2H), 2.49 – 2.36 (m, 2H), 1.98 (dt, *J* = 34.4, 7.3 Hz, 6H), 1.67 (d, *J* = 1.9 Hz, 12H), 1.36 (s, 9H), 1.30 – 1.11 (m, 4H), 0.83 (td, *J* = 7.4, 5.4 Hz, 3H). ^{13}C NMR (101 MHz, MeOD) δ 174.69 (d, *J* = 11.8 Hz), 173.09, 172.50, 170.73 (d, *J* = 4.9 Hz), 150.95 (d, *J* = 11.8 Hz), 142.87 – 141.82 (m), 140.81 (d, *J* = 4.5 Hz), 128.65, 125.85 – 124.89 (m), 123.90 (d, *J* = 3.6 Hz), 122.08, 117.28, 111.25, 102.63, 81.53, 56.93, 49.27, 47.41, 47.20, 46.98, 43.18, 31.81 (d, *J* = 11.6 Hz), 30.53, 29.58, 28.83, 26.96, 26.87, 24.28, 22.86, 22.96 – 20.99 (m), 16.99, 12.85 (d, *J* = 3.5 Hz). HRMS (ESI), m/z: [M+H]+ calcd for $C_{46}H_{64}N_4O_9S$, 848.4394; found, 848.4392.

3-(4-(2-((E)-3-((Z)-1-(6-(tert-butoxycarbonyl)-4,8,22-trioxo-26-(2-oxohexahydro-1H-thieno[3,4-d]imidazol-4-yl)-12,15,18-trioxa-5,9,21-triazahexacosyl)-3,3-dimethylindolin-2-ylidene)prop-1-en-1-yl)-3,3-dimethyl-3H-indol-1-ium-1-yl)-N-butylbutanamido)propane-1-sulfonate (14)

Compound **13** (31 mg, 36.5 µmol) was dissolved in DMF together with Biotin-PEG-3-NH_2 (22 mg, 41.3 µmol) and DIPEA (26 µL, 146 µmol). The mixture was cooled to 0°C and HATU (15.5 mg, 41.3 µmol) and HOBt (2.5 mg, 20.6 µmol) were added. The mixture was allowed to stir at room temperature for 6h (controlled by TLC). The reaction was quenched with water and the mixture was evaporated *in vacuo*. The crude product was purified by flash chromatography on silica SiO_2 gel with gradual eluting with DCM/methanol from 98:2 to 90:12 v:v%. Yield: 27 mg (59%) as a dark red solid. ^{1}H NMR (400 MHz, MeOD) δ 8.45 (t, *J* = 13.4 Hz, 1H), 7.48 – 7.39 (m, 2H), 7.38 – 7.29 (m, 4H), 7.27 – 7.16 (m, 2H), 6.57 – 6.40 (m, 2H), 4.60 (ddd, *J* = 9.8, 7.4, 5.6 Hz, 1H), 4.38 (dd, *J* = 7.9, 4.8 Hz, 1H), 4.20 (dd, *J* = 7.9, 4.5 Hz, 1H), 4.17 – 4.05 (m, 4H), 3.58 – 3.46 (m, 10H), 3.43 (t, *J* = 5.6 Hz, 4H), 3.18 – 3.05 (m, 2H), 2.86 – 2.69 (m, 3H), 2.68 – 2.57 (m, 4H), 2.55 – 2.35 (m, 3H), 2.17 – 2.07 (m, 2H), 2.06 – 1.89 (m, 6H), 1.67 (d, *J* = 1.6 Hz, 11H), 1.62 – 1.39 (m, 3H), 1.36 (d, *J* = 1.5 Hz, 9H), 1.30 – 1.11 (m,

6H), 0.83 (td, *J* = 7.4, 2.3 Hz, 3H). ^{13}C NMR (101 MHz, MeOD) δ 174.78, 174.67, 174.60, 173.14, 173.05, 172.53, 172.18, 170.65, 170.61, 170.37, 164.64, 151.02, 150.87, 142.00, 141.92, 140.85, 140.82, 128.67, 128.62, 125.37, 125.32, 122.08, 111.29, 111.21, 102.65, 102.49, 81.70, 70.19, 70.06, 69.84, 69.20, 69.11, 61.96, 60.23, 55.61, 54.68, 54.46, 50.31, 49.29, 49.26, 49.24, 48.93, 46.62, 45.65, 44.95, 43.49, 43.18, 42.41, 39.68, 39.07, 38.94, 36.97, 35.35, 31.90, 31.72, 30.55, 29.67, 29.61, 28.78, 28.37, 28.16, 28.11, 26.98, 26.91, 25.45, 24.37, 23.27, 22.99, 22.91, 22.71, 22.28, 19.85, 19.73, 12.89, 12.86, 11.77. HRMS (ESI), m/z: [M+H]+ calcd for $C_{64}H_{96}N_8O_{13}S_2$, 1248.6538; found, 1248.6518.

3-(N-butyl-4-(2-((E)-3-((Z)-1-(6-carboxy-4,8,22-trioxo-26-(2-oxohexahydro-1H-thieno[3,4-d]imidazol-4-yl)-12,15,18-trioxa-5,9,21-triazahexacosyl)-3,3-dimethylindolin-2-ylidene)prop-1-en-1-yl)-3,3-dimethyl-3H-indol-1-ium-1-yl)butanamido)propane-1-sulfonate (15)

Compound **14** was dissolved in a mixture of DCM/TFA (70:30) and the reaction was allowed to stir 4h at RT. The reaction mixture was evaporated *in vacuo* and dried under high vacuum overnight. The crude product was used in the next step without further purification. The quantity of product obtained was too small to record an NMR spectrum. The product was characterized by HRMS (ESI), m/z: [M-H_2O]+ calcd for $C_{60}H_{88}N_8O_{13}S_2$, 1174.5766; found, 1174.5775.

NHS ester preparation (MemGraft-Cy3, MemGraft-Cy5, 1, 2 and MemGraft-Cy3-biotin)

A corresponding cyanine carboxylic acid (5 mg scale) derivative (**7**, **8**, **9**, **10** and **15**) was dissolved in dry DCM (1 mL) followed by DIPEA (1.5 eq) and TSTU (1.5 eq). The reaction was maintained by stirring at room temperature for 6h. The reaction was quenched with water, DCM was added and the organic layer was washed with aqueous 5% citric acid and brine. Then, it was dried over Na_2SO_4, filtrated and concentrated under vacuum. NHS esters of cyanine derivatives (MemGraft-Cy3, MemGraft-Cy5, **1**, **2** and MemGraft-Cy3-biotin, respectively) were obtained as a fine powder and used in microscopy without any intermediate purification.

3-(N-butyl-4-(2-((E)-3-((E)-1-(4-methoxy-4-oxobutyl)-3,3-dimethylindolin-2-ylidene)prop-1-en-1-yl)-3,3-dimethyl-3H-indol-1-ium-1-yl)butanamido)propane-1-sulfonate (17)

Compound **7** (12 mg, 18 µmol) was added to a stirred solution of EDC hydrochloride (4 mg, 21 µmol) and 4-dimethylaminopyridine DMAP (1 mg, 8.2 µmol) in 2 mL CH_3CN/Pyridine (9:1). The mixture was allowed to stir for 15 minutes. Then, methanol was added (7.3 µL, 180 µmol) and the reaction was stirred for 12h. The solvent was evaporated *in vacuo* and the crude product was purified by preparative TLC (SiO_2, DCM/MeOH, 85:15 v/v%).^{1}H NMR (500 MHz, acetonitrile-d3) δ 8.47 (m, 1H), 7.50 (m 2H), 7.46-7.36 (m, 3H) 7.31-7.25 (m, 2H), 6.79 (t, *J* = 13.8 Hz, 2H), 6.44 (dd, *J* = 26.1 Hz, 14.0 Hz, 1H), 4.2 (t, *J* = 7.6 Hz, 2H), 4.13 (m, 2H), 3.62 (s, 3H), 3.55 (m, 2H), 3.42 (t, *J* = 8.2 Hz, 1H), 3.28 (t, *J* = 8.2 Hz, 2H), 3.21 (t, *J* = 8.2 Hz, 1H), 2.63 (m, 4H), 2.55 (t, *J* = 6.1 Hz, 1H), 2.46 (t, *J* = 6.1 Hz, 1H), 1.72 (s, 12H), 1.45 (td, *J* = 6.6 Hz, 10.3 Hz, 2H), 1.27 (m, 6H), 0.87 (t, *J* = 7.3Hz, 3H).

The quantity of compound **17** isolated was too low to record a ^{13}C NMR spectra with a good enough signal to noise ratio for peaks identification. The present ^{13}C NMR description has been established

based on a DEPT spectra for the most intense signals and chemical shifts indicated by the symbol * where indirectly determined from proton signal with HSQC and HMBC experiments.

^{13}C NMR (125 MHz, acetonitrile-d3) δ 174.68*, 173.29*, 173.23*, 151, 142*, 140*, 129*, 128.77, 128.66, 125.29, 125.19, 122.33, 122.30, 122.26, 116*, 111.56, 111.40, 111.16, 103, 102, 69*, 51, 48, 46, 45, 44, 43, 31, 30.58, 30.25, 30.07, 29.87, 29.23, 27.31, 27.28, 25, 22, 19, 13.24, 13.17.

HRMS (ESI), m/z: [M+H]+ calcd for $C_{39}H_{54}N_3O_6S$, 692.3728; found, 692.3728.

Cell viability assay

The MTT assay was performed to estimate the toxicity of the probe in mammalian cells. Five thousand HeLa cells were seeded per well and grown in 96-well plates. After 24 h of seeding, the cells were treated with different concentrations (0.02, 0.1, 0.5, 1, 2, and 5 µM) of the probes, respectively, and incubated for 15 min. After that, the staining solution was replaced with fresh medium and the cells were left for 24h at 37°C. At the end of this incubation, the medium was aspirated from all the wells, and the cells were incubated further in fresh growth medium with 0.5 mg mL^{-1} of MTT for 4 h. The medium containing MTT was then removed, after which DMSO was added to solubilize the formazan. The absorbance values were recorded using a TECAN Spark plate reader at 570 nm. The cell viability was calculated as the absorbance with respect to the positive control as a reference, in which the cells were treated with the solvent (DMSO) alone. In the negative control used as a cytotoxicity reference, cells were treated with Triton X-100 1% for 1h. The results presented here are a mean of 8 experiments ± standard deviation.

Model membrane conjugation assay

Large Unilamellar Vesicles (LUVs) were prepared by the following procedure. A stock solution of the corresponding lipids (DOPC and DOPE, 1/1, mol/mol) in chloroform was placed into a round-neck flask, after which the solvent was evaporated in vacuo. PBS was added so that the final lipid concentration was 1 mM and the mixture was sonicated. The obtained suspension of multilamellar vesicles was extruded using a Lipex Biomembranes extruder (Vancouver, Canada). The size of the filter was first 0.2 µm (7 passages) and thereafter 0.1 µm (10 passages). This generates monodisperse LUVs with a mean diameter of 0.11 µm as measured by dynamic light scattering method with a Malvern Zetasizer ZSP (Malvern Instruments S.A.). LUVs were labeled by the addition of the DMSO stock solution of the MemGraft-Cy3 probe to a final concentration of 250 µM in PBS, and incubation with LUVs for 20 min using an Eppendorf Thermomixer© C, with a rotation of 800 rpm at room temperature. The labeled liposomes were separated from the unreacted probe using a disposable size exclusion chromatography column, Illustra™, NAP™-5 from Cytiva and the fraction containing LUVs was analyzed using a MALDI-TOF MS system (Microflex ® LRF, Bruker). The optimal conditions for observation of phospholipid derivatives were found as follows: matrix DCTB in chloroform in Reflector Negative ion mode.

Protein extraction

U87 cells were seeded at a density in 5×10^6/well in a petri dish 24h before the experiment. The attached live cells were washed once with PBS. After that, 5 mL of a corresponding dye (MemGraft-

Cy3 or MemGraft-Cy3M) solution at 1 µM in HBSS was added and the cells were incubated for 10 min at room temperature. Then, the attached cells in dishes were washed with PBS and put in growth media. The cells were resuspended in the growth media by scraping the cells off the surface of the plate with a cell scraper. The cell suspension was centrifuged at 300 × g for 5 minutes. The cell pellet was washed with 3 mL of Cell Wash solution from the Mem-PER™ Plus Membrane Protein Extraction Kit from ThermoFisher, and centrifuged at 300 x g for 5 minutes. The supernatant was removed and the cells were resuspended in 1.5 mL of Cell Wash solution and transferred to a 2 mL centrifuge tube. The cells were centrifuged at 300 x g for 5 minutes and the supernatant was removed. 0.75 mL of Permeabilization Buffer from the same kit were added to the cell pellet and the mixture was vortexed. The suspension was incubated 10 minutes at 4°C under constant mixing while permeabilization occurs. The permeabilized cells were centrifuged for 15 minutes at 16.000 x g to separate cytosolic proteins. The supernatant with cytosolic proteins was entirely transferred to a new tube and stored in the permeabilization buffer. 0.5 mL of Solubilization Buffer from the same kit was added to the pellet and the cells were resuspended by pipetting up and down, and incubated at 4°C for 30 minutes with constant mixing. The suspension was then centrifuged at 16.000 x g for 15 minutes at 4°C. The supernatant containing membrane proteins and solubilized membranes was transferred to a new tube.

Protein concentration assay

Protein contents of extracted protein samples from MemGraft treated U87 cells were checked using colorimetric BioRad DC protein Assay based on Lowry method following supplier's microplate assay protocol. The tests have been performed in transparent polystyrene 96-wells microplates without lid and absorbance measurements at 750 nm done using a Tecan Spark microplate reader. A standard curve has been established using BioRad quick start BSA 7 standards set from 0.125 to 2.0 mg/mL. Typical protein concentrations obtained in extracted samples were close to 0.5 µg/mL.

SDS-Page

Purified membrane proteins in solubilization buffer and cytosolic proteins in permeabilization buffer were directly diluted with 4x Laemmli sample buffer (BioRad) and gels (BioRad Criterion 4-20%, 1mm TGX 18-wells precast Gels) were loaded with 30 µL/wells. BioRad Precision Plus Protein™ All Blue Prestained Protein Standards (10-250 kD) was used as ladder. Resulting gels were run in 1x Tris-Glycine Buffer pH = 9 (Euromedex) with 0.2 % SDS added with BioRad Powerpac generator (150 V, 300 W) for about 35 min. After SDS-Page migration, a fluorescence image of the gel were took using Syngen G:Box Chemi XRQ (Ex: Green LED 520-550 nm Em: 605 nm filter). After that, gels were stained with Coomassie solution overnight and then destained using successive wash in 1 % Acetic acid baths until non-specific staining was completely removed.

Additional experimental data

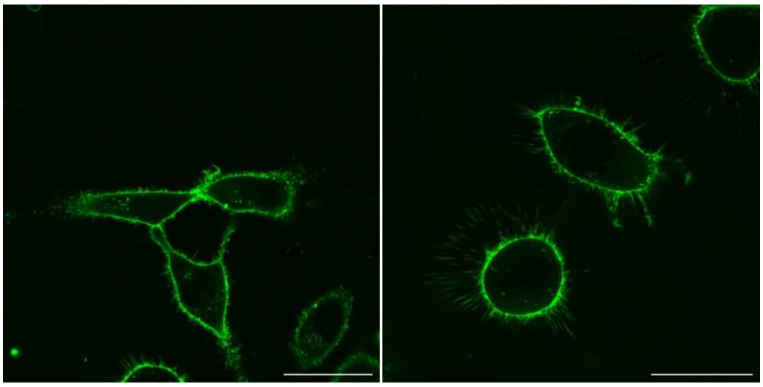

Figure S2. Fluorescence imaging of HeLa cells labelled with MemGraft-Cy3. Dye concentration was 500 nM. Scale bar: 30 µm.

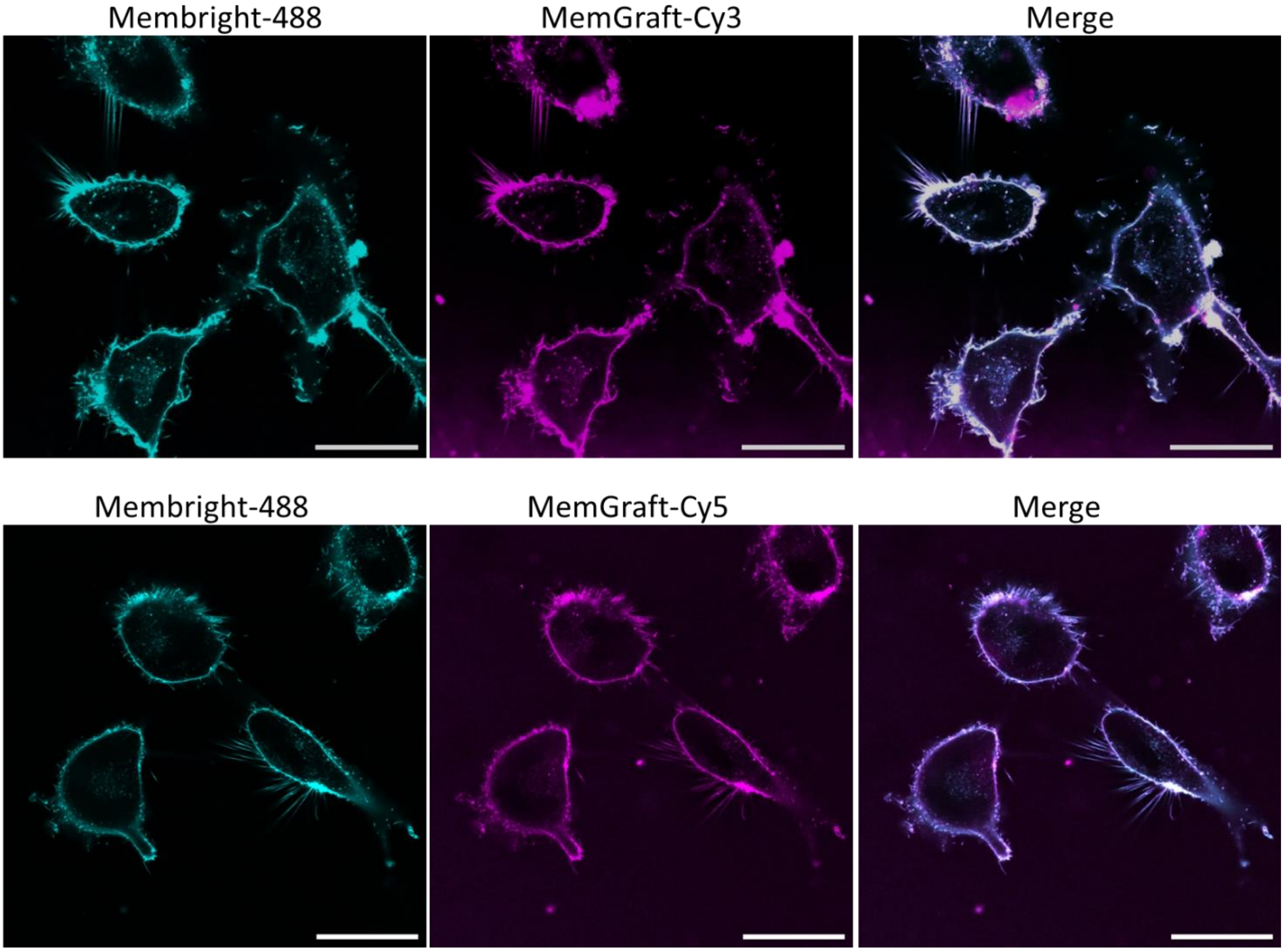

Figure S3. Fluorescence imaging of U87 cells labelled with MemGraft-Cy3 and MemGraft-Cy5 in comparison to MemBright-488. Left panels: MemBright-488, middle panels: MemGraft-Cy3 and MemGraft-Cy5; right panels: merged images. Colocalization Pearson's coefficients are 0.84 and 0.85 for MemGraft-Cy3 and MemGraft-Cy5, respectively. Dye concentrations: 500 and 200 nM for MemGraft and MemBright-488, respectively. Scale bar: 30 µm.

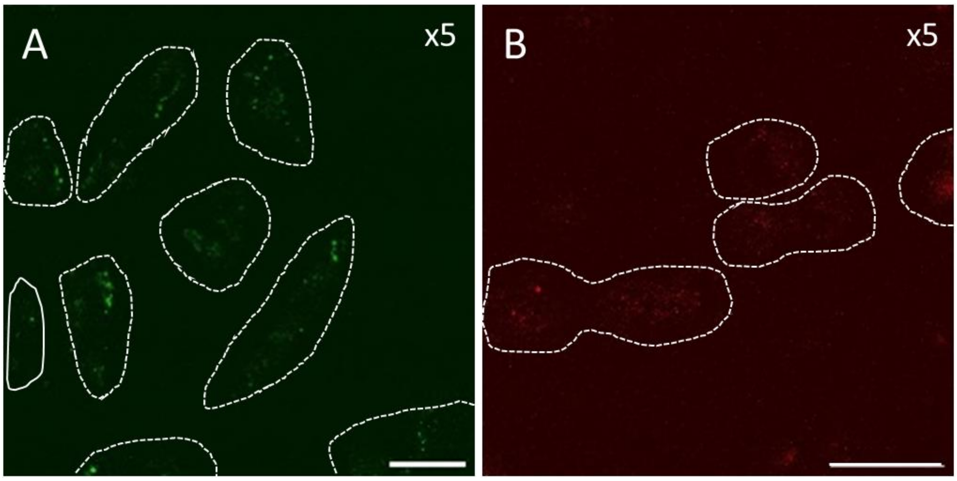

Figure S4. Fluorescence labeling of plasma membranes using non-activated carboxylic acid analogues **7** (A) and **8** (B). Confocal fluorescence microscopy of U87 cells incubated for 5 min with the dyes at 1 µM concentration. Scale bar: 30 µm. These are images from Figures 2B and 2D, respectively, with 5-fold multiplied intensity. The cells are framed by a dotted contour.

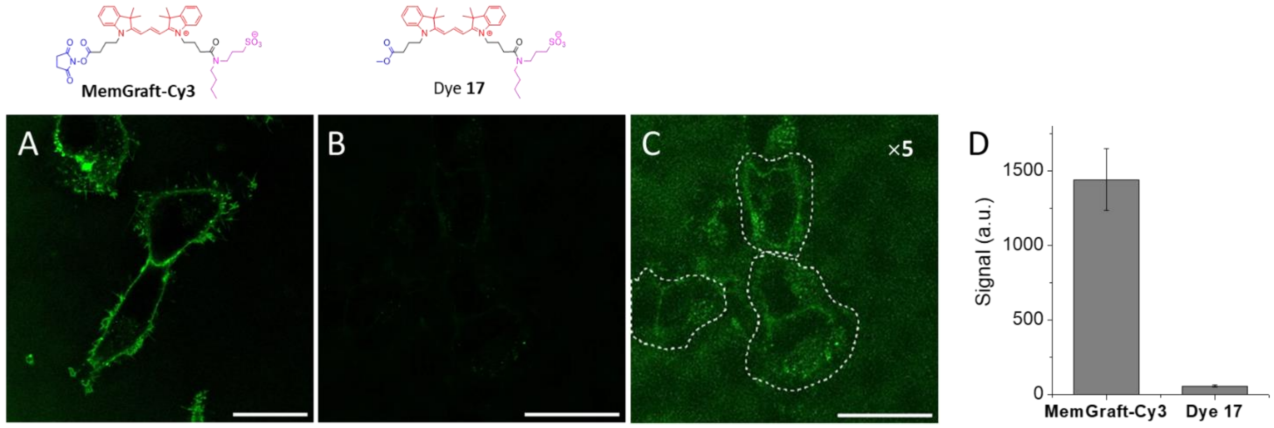

Figure S5. Fluorescence labeling of plasma membranes using MemGraft-Cy3 (A) and control analogue methyl ester **17** (B). Confocal fluorescence microscopy of U87 cells incubated for 5 min with the dyes at 0.5 µM concentration for both conditions. Scale bar: 30 µm. (C) The same image as (B) with 5-fold multiplied pixel intensity to show the lack of labeling. The dashed contours highlight the cells. (D) Quantitative image analysis: fluorescence signal at the plasma membrane (fluorescence intensity minus background intensity) for the conditions of panels A and B. Four regions of interest were analyzed per condition. The errors are the standard deviation of the mean.

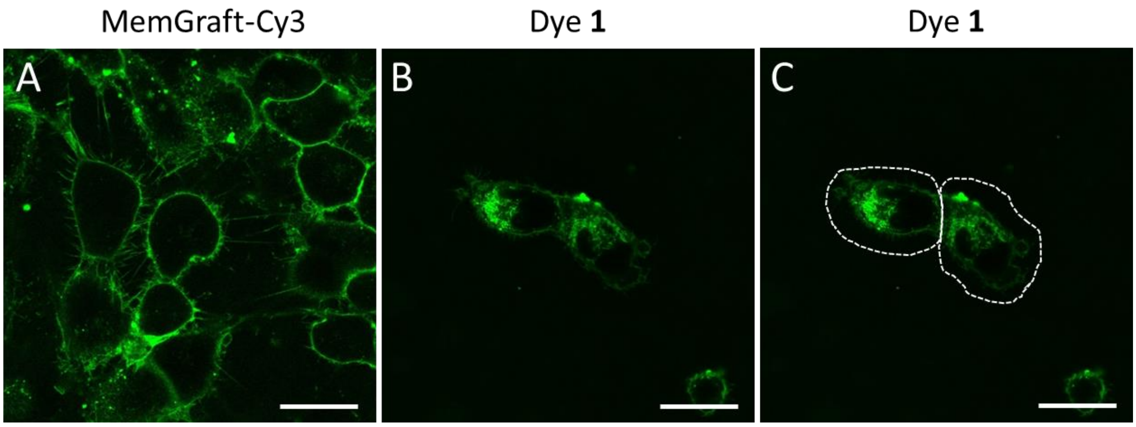

Figure S6. Comparison of MemGraft-Cy3 dye (A) with a control dye **1** without butyl anchor (B, C) at 100 nM concentration. Scale bar: 30 µm. (C) The same image as (B) with dashed contours highlight the cells.

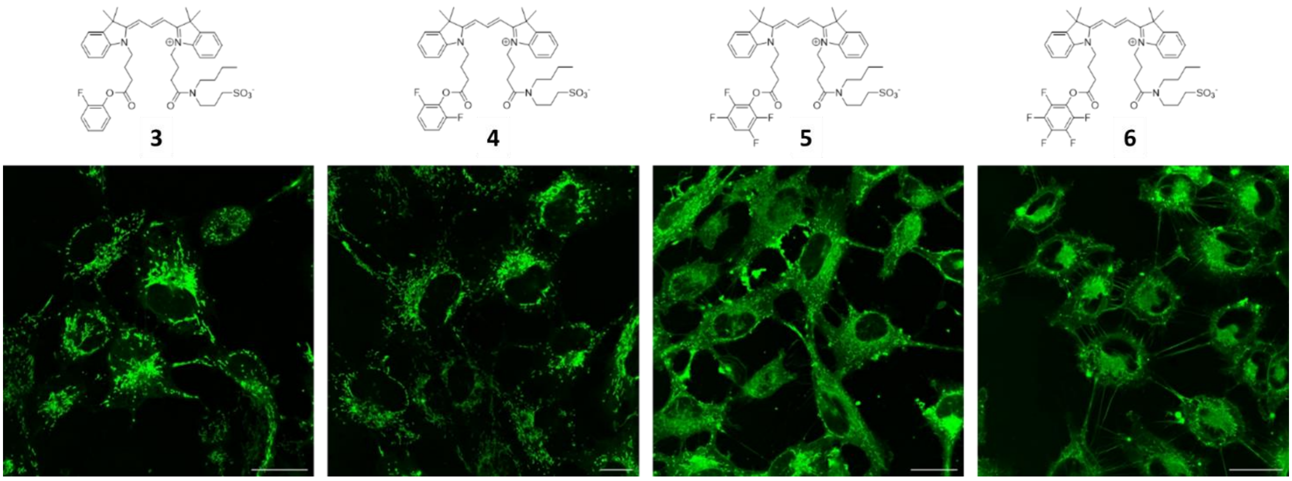

Figure S7. Fluorescence labelling of U87 cells with dyes **3**-**6** after 15 min incubation and washing. Dye concentrations: 500 nM. Scale bar: 30 µm.

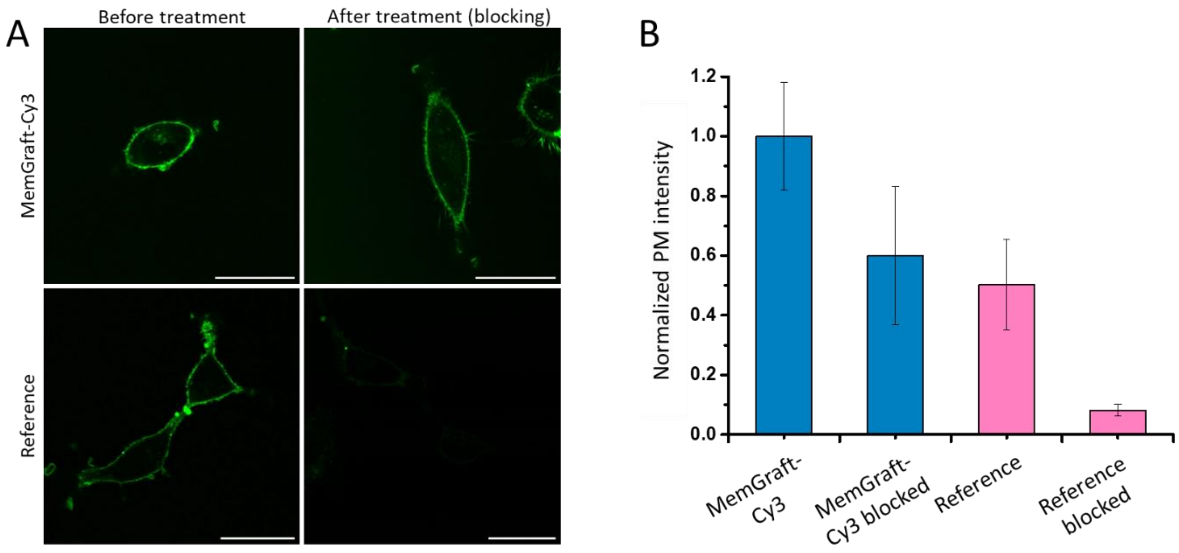

Figure S8. (A) Confocal images of U87 cells incubated with MemGraft-Cy3 and reference dye disulfo-Cy3-NHS before and after pretreatment with Sulfo-NHS-acetate (blocking of PM labelling). (B) Fluorescence intensity of cells labelled with MemGraft-Cy3 and a reference dye (disulfo-Cy3-NHS) without and with pre-incubation with 1 mM of Sulfo-NHS-Acetate for 60 min at 37°C. The concentration of the dyes was 500 nM. The incubation time was 5 min. The errors are the standard deviation of the mean based on ~20 cells.

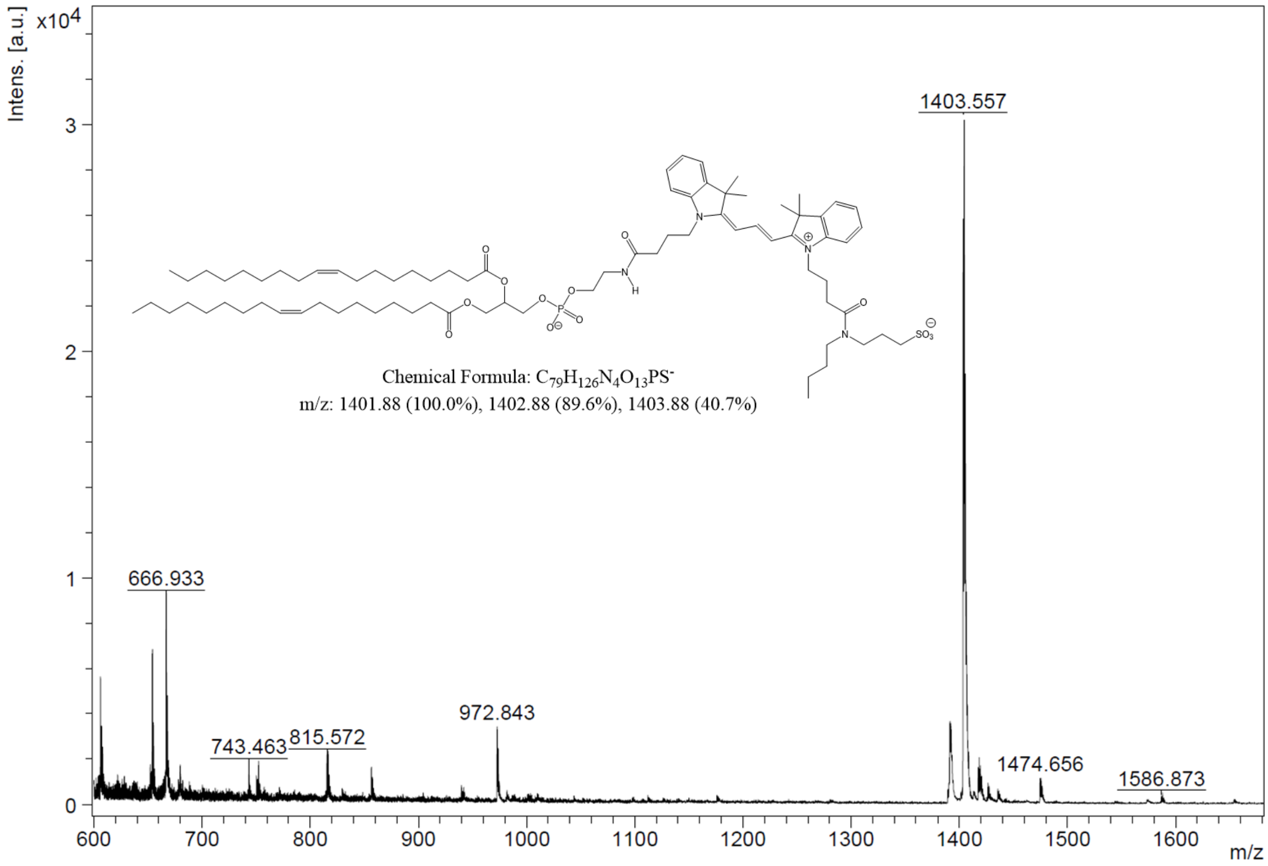

Figure S9. Mass analysis of expected conjugate of MemGraft-Cy3 with DOPE lipid after reaction of the probe with liposomes composed of DOPC/DOPE mixture, 1/1, mol/mol.

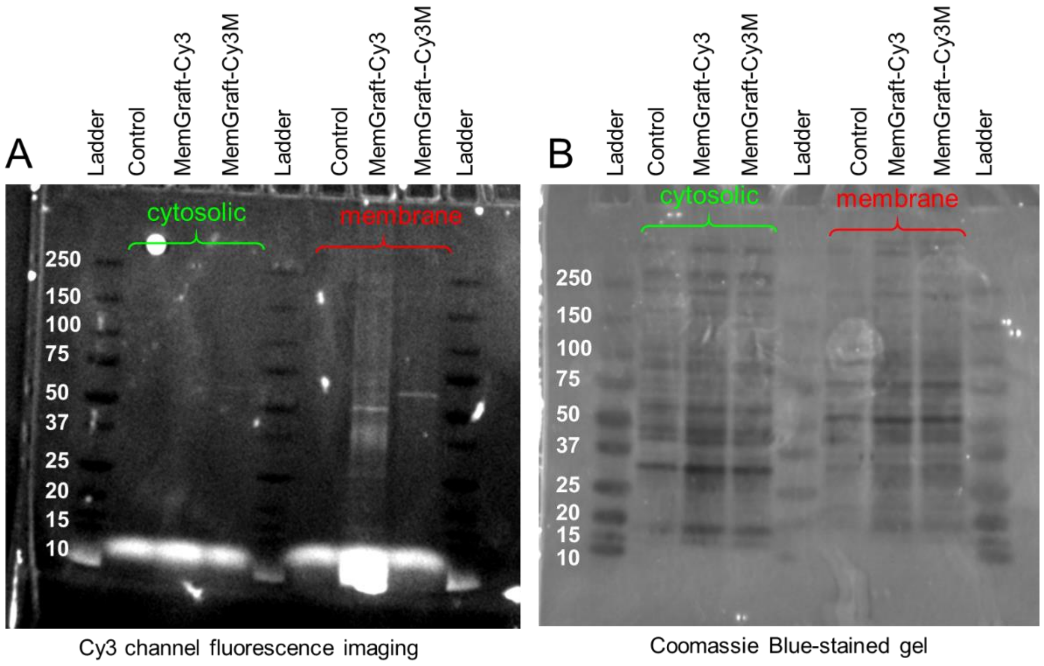

Figure S10. SDS-polyacrylamide gel electrophoresis (SDS-PAGE) results of cytosolic and membrane proteins extracted from U87 cells stained with MemGraft-Cy3, MemGraft-Cy3M or without staining (control). (A) fluorescence imaging of the gel with Cy3 channel. (B) Brightfield image of the Coomassie Blue-stained gel.

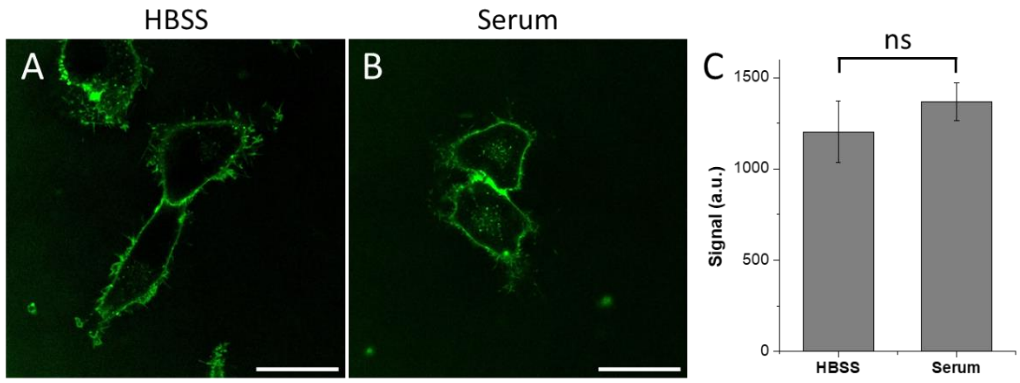

Figure S11. Effect of addition of serum on the fluorescence labeling of plasma membranes with MemGraft-Cy3. (A) Confocal fluorescence microscopy of U87 cells labelled with MemGraft-Cy3 at 0.5 µM concentration. (B) Imaging of the same sample of labelled cells after 10 min of incubation with 20% FBS (serum) in HBSS. Scale bar: 30 µm. (C) Quantitative image analysis: fluorescence signal at the plasma membrane (fluorescence intensity minus background intensity) for the conditions of panels A and B. Four regions of interest were analyzed per condition. The errors are the standard deviation of the mean. The difference in panel B is not significant (ns) according to one-way ANOVA statistics test (significance criteria $p < 0.05$).

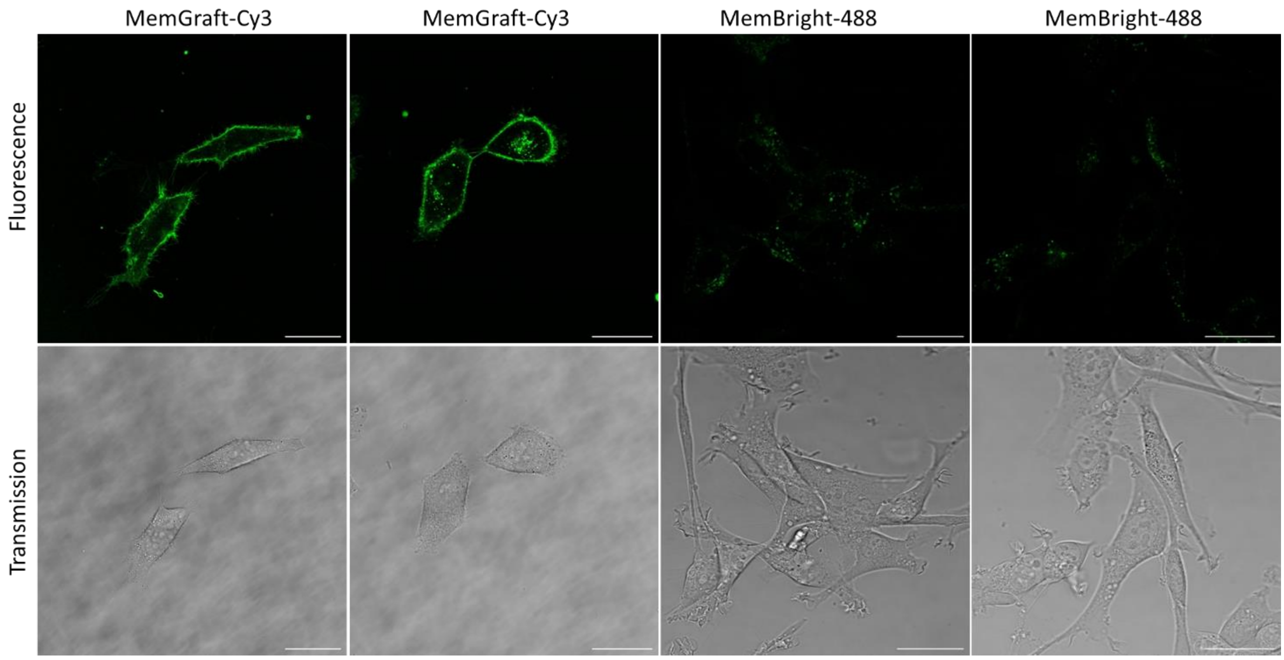

Figure S12. Fluorescence imaging of U87 cells labelled with MemGraft-Cy3 and MemBright-488 after trypsinization of seeding to a new microscopy plate and incubation for 24h in the full growth medium with 20% FBS (serum). Concentration of MemGraft-Cy3 and MemBright-488 were 1 and 0.2 µM, respectively. The results are shown in duplicates for each condition. Scale bar: 30 µm.

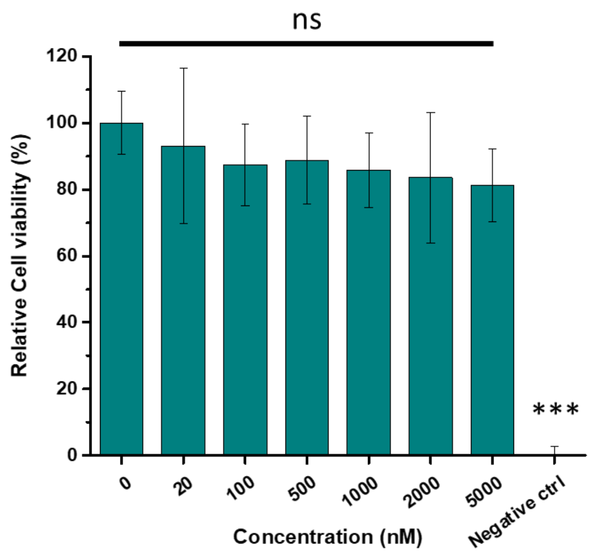

Figure S13. Viability of Hela cells after incubation with MemGraft-Cy5 at different concentrations for 24h. Negative control (to achieve maximum toxicity) was treatment of cells with 1% Triton X-100 for 1h. Data for MemGraft-Cy3 are not available because of potential cross-talk of the dye with MTT assay. Statistics analysis by one-way ANOVA with Dunnet test pair comparison with 0 for $P< 0.05$. *** $p < 0.001$.

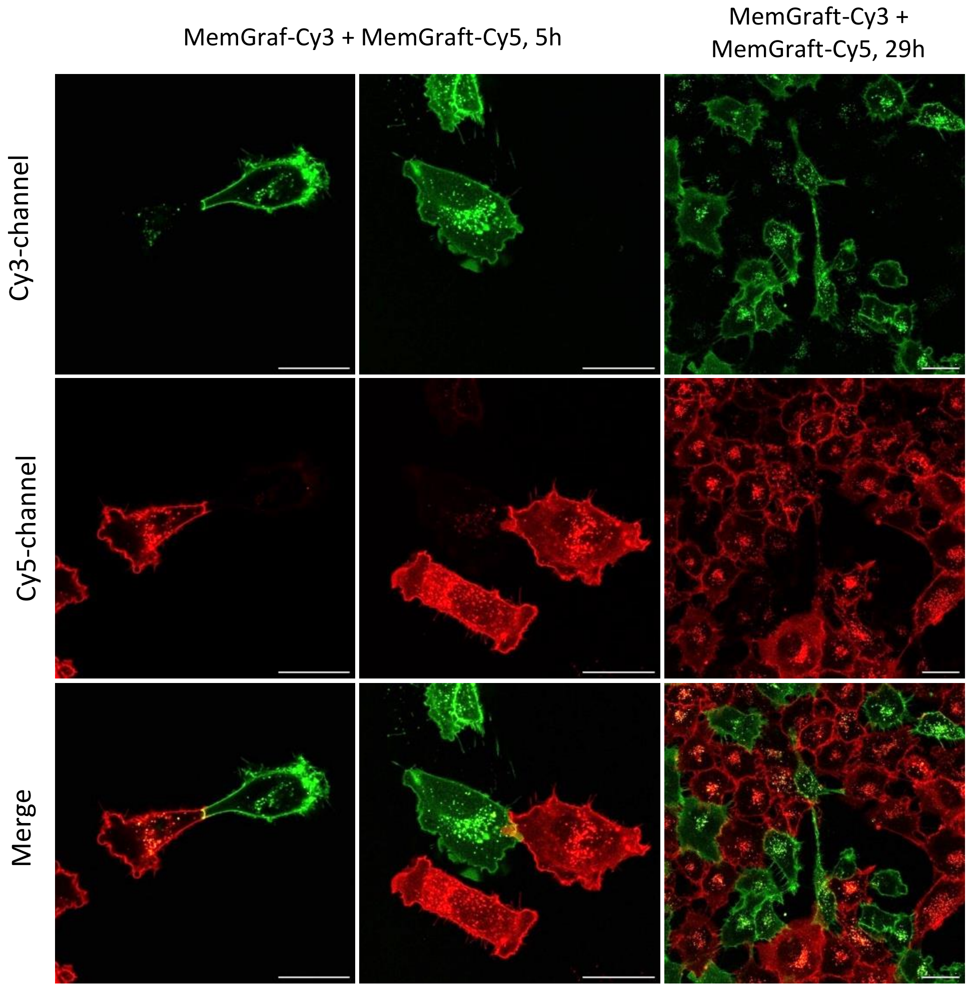

Figure S14. Additional examples of co-seeding of cells stained with MemGraft-Cy3 and MemGraft-Cy5 (C): after 5h ; (D): after 29h. Cells stained only with MemGraft-Cy3 or MemGraft-Cy5 are shown respectively in A and B Scale bar: 30 μm.

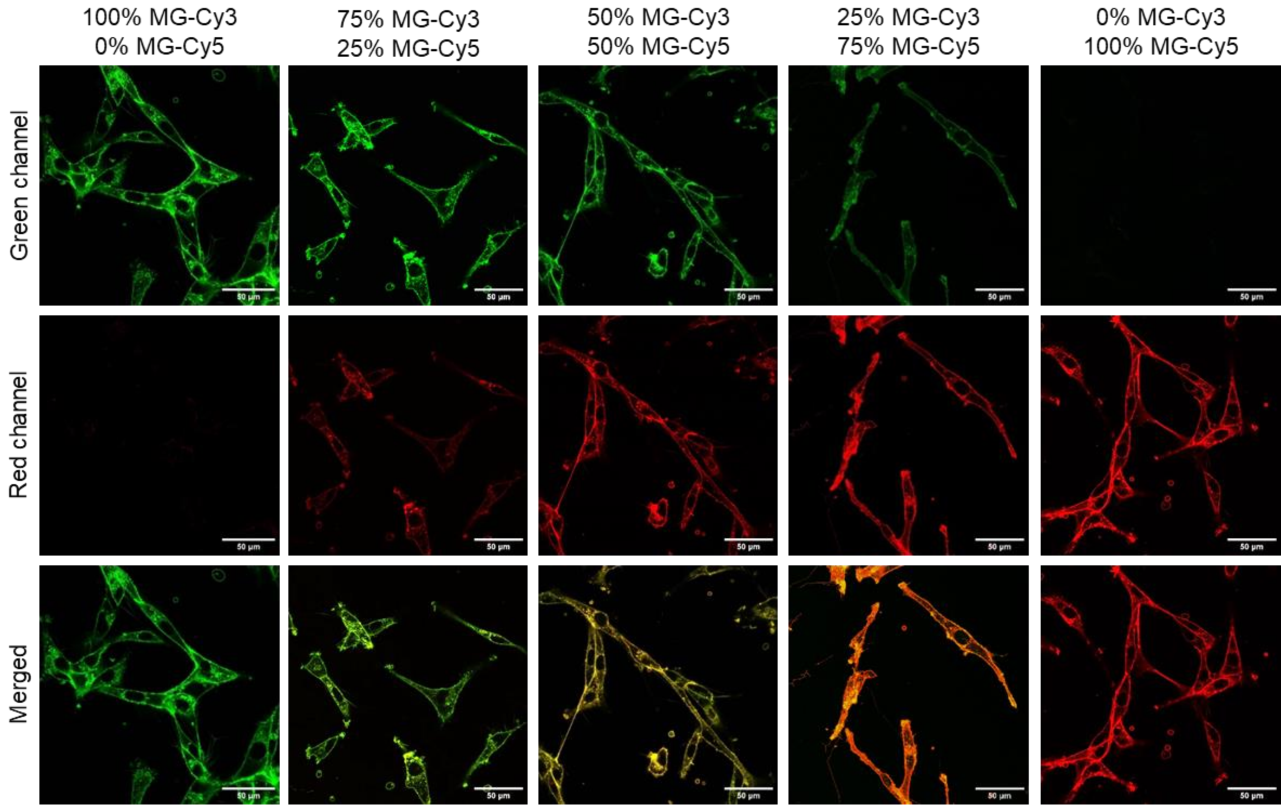

Figure S15. Confocal fluorescence microscopy of U87 cells stained with varied molar % of MemGraft-Cy3 (MG-Cy3) / MemGraft-Cy5 (MG-Cy5): 100%/0%, 75%/25%, 50%/50%, 25%/75%, 0%/100% (left to right). Upper panels: green channel (Cy3). Middle panels: red channel (Cy5). Lower panels: Merged channels. Total dye concentration was 1 μm in each case. Scale bars: 100 μm.

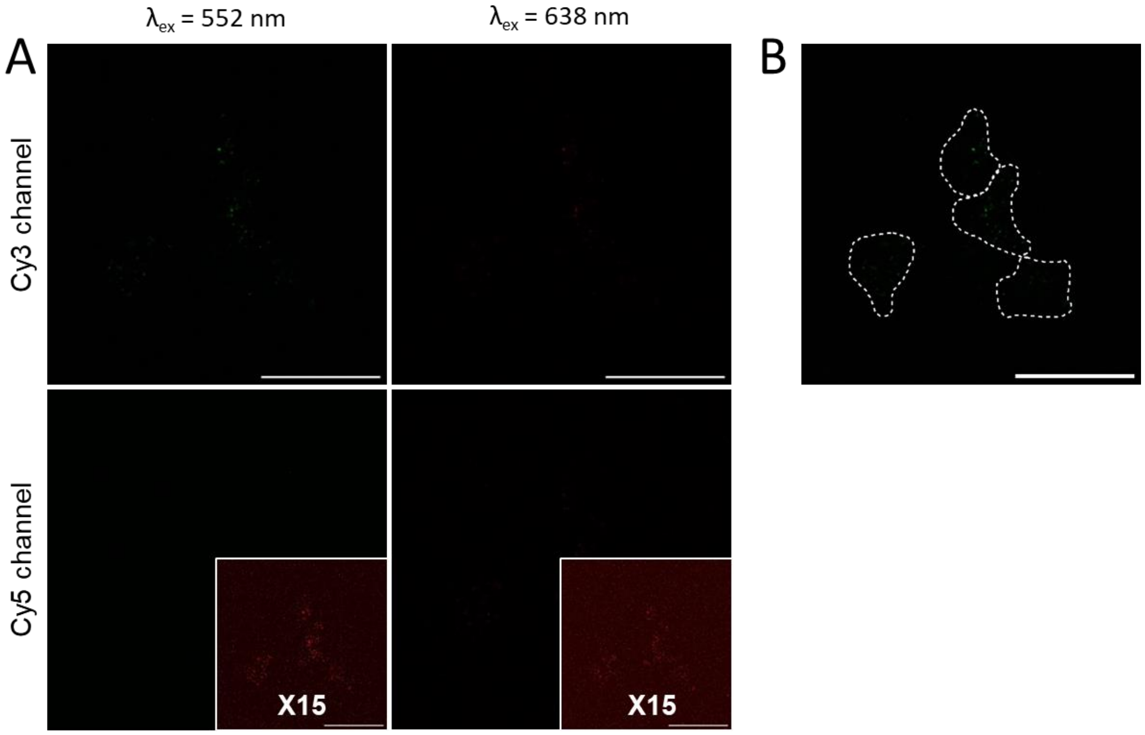

Figure S16. (A) Confocal fluorescence microscopy of U87 cells incubated for 30 min with streptavidin-Cy5 adduct only as a control. On the left panel, the images are recorded with excitation of Cy3 (552 nm). On the right panel, the images are recorded with excitation of Cy5 (638 nm). Upper panels: Cy3 channel (excitation at 552 nm). Lower panels: Cy5 channel (excitation at 638 nm). Final streptavidin-Cy5 concentration was 0.1 mg/mL Scale bars: 30 μm. (B) To indicate the presence of the cells, they are surrounded by a dotted contour (Cy3 channel with 552 nm excitation was used).

Supporting videos

Video S1. Fluorescence video imaging of U87 cells labelled with MemGraft-Cy3. Recording time was 3 hours with 1 frame / min. Dye concentration was 1 µM. Scale bar: 50 µm.

Video S2. Fluorescence video imaging of co-seeded U87 cells labelled with MemGraft-Cy3 (green) and MemGraft-Cy5 (red). Recording time was 1 hour with 1 frame / min. Dye concentration was 1 µM. Scale bar: 50 µm.

References

(1) Aparin, I. O.; Yan, R.; Pelletier, R.; Choi, A. A.; Danylchuk, D. I.; Xu, K.; Klymchenko, A. S. Fluorogenic Dimers as Bright Switchable Probes for Enhanced Super-Resolution Imaging of Cell Membranes. *J. Am. Chem. Soc.* **2022**, *144*, 18043-18053.
